# Reactive Active Learning: An Efficient Approach for Training Machine Learning Interatomic Potentials for Reacting Systems

**DOI:** 10.1021/acs.jctc.5c00920

**Published:** 2025-09-03

**Authors:** Siddarth K. Achar, Priyanka B. Shukla, Chinmay V. Mhatre, Leonardo Bernasconi, Caitlyn Y. Vinger, J. Karl Johnson

**Affiliations:** † Computational Modeling & Simulation Program, 6614 University of Pittsburgh, Pittsburgh, Pennsylvania 15260, United States; ‡ Department of Chemical & Petroleum Engineering, University of Pittsburgh, Pittsburgh, Pennsylvania 15261, United States; § Center for Research Computing and Data, University of Pittsburgh, Pittsburgh, Pennsylvania 15213, United States; ∥ Department of Electrical & Computer Engineering, University of Pittsburgh, Pittsburgh, Pennsylvania 15261, United States; ⊥ Department of Chemistry, University of Pittsburgh, Pittsburgh, Pennsylvania 15260, United States

## Abstract

Discovering chemical reaction pathways using quantum mechanics is impractical for many systems of practical interest because of unfavorable scaling and computational cost. While machine learning interatomic potentials (MLIPs) trained on quantum mechanical data offer a promising alternative, they face challenges for reactive systems due to the need for extensive sampling of the potential energy surface in regions that are far from equilibrium geometries. Unfortunately, traditional MLIP training protocols are not designed for comprehensive reaction exploration. We present a reactive active learning (RAL) framework that is designed to efficiently train MLIPs to achieve near-quantum mechanical accuracy for reactive systems for situations where one may not have prior knowledge of the possible transition states, reaction pathways, or even the potential products. Our method combines automated reaction exploration, uncertainty-driven active learning, and transition state sampling to build accurate potentials. We demonstrate RAL’s effectiveness across three different systems: uncatalyzed ammonia synthesis (gas-phase), methanimine hydrolysis (solution phase), and methane activation on titanium carbide surfaces (heterogeneous). The resulting MLIPs accurately predict reaction barriers and transition states. For catalysis, we show that RAL-trained MLIPs identify Ti_2_C as the most active methane activation surface (90% decomposition at 1000 K) through C-vacancy mediated mechanisms. The framework’s ability to simulate large systems (∼900 atoms) over nanosecond time scales provides previously inaccessible insights into surface poisoning and reaction networks. We show that reactive exploration is essential for adequately capturing the potential energy surface, with chemical and configurational sampling working synergistically to improve model accuracy. Our results establish general guidelines for training robust reactive potentials and open new possibilities for computational discovery of catalysts and reaction mechanisms.

## Graphical abstract

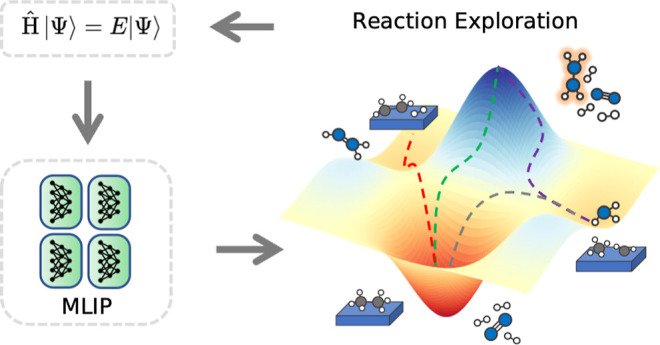

## 1 Introduction

Modeling chemical reactions with precision hinges crucially on the detailed representation of the potential energy surface (PES), which encapsulates the energy landscapes over which chemical reactions occur. An effective PES representation must capture a sufficient breadth of both conformational (inter- and intramolecular interactions) and chemical events. The chemical event PES includes transition states, bond formation, bond breakage, and the different types of reactants and products.

Traditional exploration of this reactive space employs quantum mechanical approaches, such as density functional theory (DFT), coupled with transition state (TS) finding tools, rare event sampling methods, or molecular dynamics (DFT-MD or AIMD) calculations. While relatively accurate, these methods are very computationally intensive and limited to small system sizes, typically no more than a few hundreds of atoms, and time scales limited to picoseconds.

While classical potentials are computationally inexpensive, they suffer from significant drawbacks, including poor accuracy, limited transferability, and a fixed bonding topology that fails to model the dynamic nature of chemical bonds. Empirical reactive potentials, like ReaxFF,^1,2^ are able to capture bond breaking and formation events, but are often insufficiently accurate for reliable prediction of transition states.

In response to these challenges, machine learning interatomic potentials (MLIPs) have emerged as a versatile alternative. These models are flexible and use learnable functions to accurately map molecular structures onto complex PESs. They employ molecular descriptors that encode reaction specifics without the explicit addition of bonding terms, which are applicable across various scenarios, from solid-state physics to solution-phase reactions. Application of MLIPs include materials,^3^ molecular,^4^ biological,^5^ and heterogeneous systems.^6,7^ However, MLIPs are not without limitations. A notable concern is that they lack any description of the underlying physics and chemistry. This means that extrapolation outside their training data can lead to unphysical predictions and large errors in regions of the PES that are not adequately explored. To mitigate this, conventional frameworks for training MLIPs often employ active learning (AL)—an iterative and model-aware data generation strategy designed to minimize data set size while maximizing coverage of uncertain regions. A common implementation of AL is to use MLIP-driven molecular dynamics (MD) simulations to explore the PES and identify high-uncertainty configurations.^8^ However, because MD trajectories sample configurations from the Boltzmann distribution, they tend to remain near equilibrium regions of the PES. As a result, bond-breaking or bond-forming events are rare events, unless the temperature is extremely high. This sampling bias can limit the ability of MLIPs to accurately model reactive processes unless such configurations are explicitly included during training. This underscores the critical need for efficient and sufficiently comprehensive training data generation strategies to enable MLIPs to accurately model chemical reactions.

Many researchers have developed methods for training MLIPs to account for chemical reactions.^7,9–24^ We briefly summarize several of these approaches here in order to compare and contrast with our formalism.

Young and co-workers^10^ developed a data-efficient method of training MLIPs that only requires hundreds to thousands of energy and force evaluations with the reference method. They demonstrated the effectiveness of their approach by computing reaction PESs for both gas-phase and liquid phase reactions, including S_N_2 substitutions and several Diels–Alder reactions.^10,11^ Their method uses a hierarchical and AL approach in conjunction with a new error metric and requires a priori knowledge of the reactions to be modeled, such as the TS geometries.

Several researchers^12–18^ have used enhanced sampling techniques, metadynamics,^15,25^ umbrella sampling,^26^ transition path sampling,^27^ uncertainty-driven dynamics,^28^ and on-the-fly probability enhanced sampling^29^ as tools in the training of MLIPs to account for specific chemical reactions. Using enhanced sampling methods requires defining reaction coordinates or collective variables, which are used to drive the evolution of the system and explore predefined reaction pathways. Hence, one needs detailed knowledge of the reactions to apply these methods.

Schaaf et al.^19^ focused on the minimum energy path for CO_2_ reduction on cubic In_2_O_3_, employing an AL training protocol with MD and geometric relaxation. In their approach, the final step of AL utilized the nudged elastic band (NEB)^30–32^ method. This required that the reactants and products were predetermined, limiting the exploration of reaction pathways with nonpredetermined products.

Guo et al.^23^ introduced a curriculum-based training approach for methane activation on [CuOCu]^2+^ sites in zeolites. A key feature of their method is the screening of descriptors to identify representative sites for reactions, which they explored using extensive DFT-NEB calculations for initial data generation. While their pipeline is tailored to a specific system, the curriculum-based training framework could, in principle, be adapted to other reactions with appropriate system-specific modifications.

Zhang et al.^20^ applied the “Nanoreactor” formalism to explore chemical reactions naturally within an MD simulation. This method involves performing long MD simulations varying the temperature and density of the system according to a program to enhance the probability of reaction events. It has the advantage of not requiring a priori knowledge of the chemical reactions. Similarly, Zeng et al.^21^ studied methane oxidation using DeePMD^33^ by exploring chemical reactions through very high temperature MD simulations with the empirical force field ReaxFF. This same approach was also used to develop a reactive potential for *n*-dodecane pyrolysis.^22^ However, using ReaxFF or some other empirical potential model to generate training data has significant drawbacks. First, such models are not generally available for use and require significant training to develop. Second, the accuracy of ReaxFF does not approach DFT accuracy. This means that ReaxFF may generate unphysical structures that could impact the MLIP training. Yoo et al.^34^ also trained a reactive neural network force field for CHNO systems by running high-temperature MD with ReaxFF to generate initial configurations. They further refined their methodology by performing MD-AL loops, relying on high temperature MD to sample reactions. However, the authors did not use an uncertainty-driven approach to meaningfully add configurations to the training data set but rather the convergence was dependent on the root-mean-square error (RMSE) of fixed test sets.

Schreiner and co-workers^35^ introduced the Transition1x data set containing 9.6 million DFT data points around reaction pathways computed at the ωB97X^36^/6–31G­(d) level of theory. The reactive intermediates were generated by running DFT-NEB on 10,000 organic reactions. The PaiNN^37^ architecture was trained and evaluated on Transition1x, QM9x, and ANI1x data sets. The authors achieved lower RMSE for predicting unseen transition state energies and forces for models trained on Transition1x data set, highlighting Transition1x as a crucial resource for developing MLIPs capable of accurate reaction barrier and transition-state predictions.

Lee et al.^24^ developed a method to train reactive MLIPs by automating the sampling reaction pathways through the use of a graph enumeration algorithm^38^ in conjunction with the single-ended growing string method (SE-GSM)^39^ for transition state finding. The SE-GSM method does not require knowledge of the product geometries; it only requires the geometry of the reactant molecules and a list of bonds to add and break to achieve a desired change in the connectivity of the reactants to form the products. These add/break commands are the driving coordinates that create new structures along the reaction pathway. Lee et al.^24^ used the GFN2-xTB^40^ tight-binding method to generate the reaction pathways with SE-GSM. They then filtered out reaction pathways and refined the PES by performing NEB transition state searches with GFN2-xTB. Finally, they filtered the NEB data and recalculated the energies and forces using DFT at the ωB97X^36^/6–31G­(d) level of theory. They also included normal mode sampling to explore equilibrium structures.

We have developed a new approach for efficiently and accurately training MLIPs for chemically reactive systems. Our goal is to limit the need for human intuition, making it as automated as reasonably possible. Our approach employs a query-by-committee (QBC)^41^ strategy, using uncertainty among MLIP predictions to iteratively improve the MLIPs through AL, but doing so through explicit and automatic exploration of chemical reactions. Hence, we call our method reactive active learning (RAL). Our approach eliminates the need to specify predefined reaction pathways and collective variables, which require human intuition and introduce bias into the training. Instead, we only require the user to specify a set of reactant molecules and allow RAL to identify potential reaction intermediates and products. Algorithms used to dynamically enumerate these products during training are discussed in the Methods section. For homogeneous systems we use the SE-GSM method to eliminate the need to specify product geometries. For heterogeneous surface reactions we utilize the NEB method because SE-GSM is not currently applicable to heterogeneous reactions.

## 2 Methods

### 2.1 Reactive Active Learning Workflow

The overall schematic of our RAL workflow is shown in Figure 1a. This workflow is similar in spirit to other QBC schemes, like DP-GEN,^8^ where the configuration exploration tool typically used is MD. In contrast, RAL is designed to explicitly sample reaction mechanisms by applying QBC to TS finding tools. In this section, we explain the techniques that we used to train MLIPs for two uncatalyzed gas/condensed phase systems (N_2_/H_2_ and CH_2_NH/H_2_O) and heterogeneous catalytic systems (Ti_*x*_C_*y*_/CH_4_). We provide detailed explanations of the methods used in each test case in later sections.

**Figure 1:**
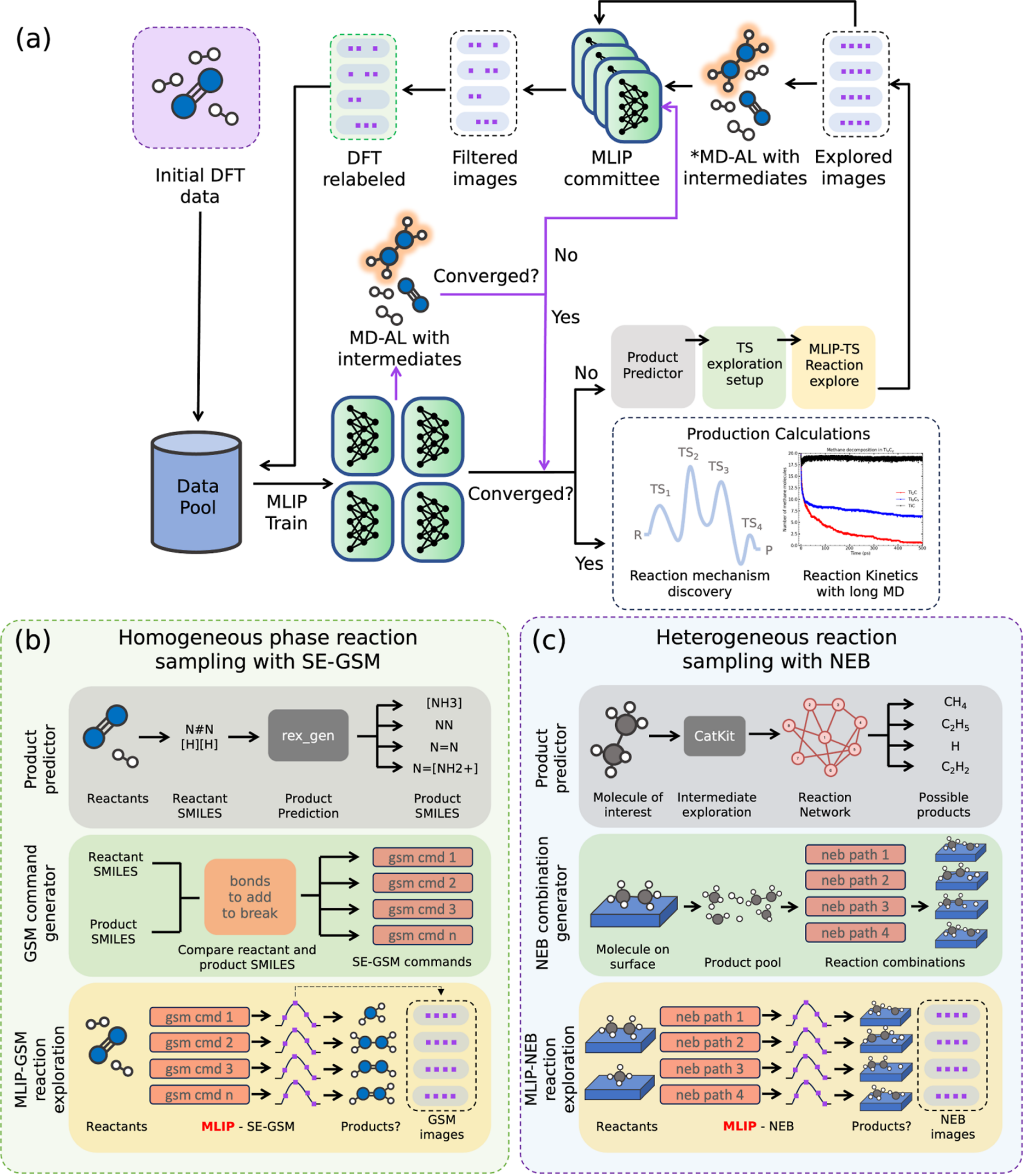
(a) Reactive active learning workflow schematic. Initial data are collected to generate bootstrap MLIPs. The committee of MLIPs is used to perform reaction exploration through three steps: product predictor, TS exploration setup, MLIP-GSM reaction exploration. Explored images from this step are filtered using the committee of MLIPs and are DFT relabeled based on a user-defined upper and lower threshold of maximum force deviation. Relabeled images are added back to the training data for the next iteration of RAL. SC-AL cycles employ MD-AL steps depicted with purple lines. NSC-AL uses a single MD-AL step denoted by * in the Figure. (b) Schematic of the homogeneous phase reaction exploration step. Product predictor: Graph2SMILES generates products. GSM command generator: reactant and product SMILES are compared to generate a list of SE-GSM add and break commands. MLIP-GSM reaction exploration: reaction paths and TS states are generated with SE-GSM. Intermediates and products are filtered and relabeled. (c) Schematic of the heterogeneous phase reaction exploration setup. Steps are similar to the homogeneous phase, but using CatKit for product generation and NEB for reaction path prediction.

The RAL workflow consists of several steps. At the heart of the method is the RAL step, which involves AL of reaction pathways. This RAL step will discover new molecules that have not been seen by the MLIP. This necessitates the addition of an MD-AL step within the RAL workflow, exploring configuration space of these new species. Please note the distinction between the RAL step and the MD-AL step, both of which are part of the RAL workflow shown in Figure 1. A summary of the general steps in the RAL workflow is given here. 1. Select a set of molecules to use as a bootstrap data set. Note that this set does not need to include intermediates or reaction products, as these will be discovered during RAL. This is a significant advantage of RAL because, in general, one may only know the initial reactants and will not have detailed knowledge of the possible intermediates and products. 2. Generate a committee of bootstrap MLIPs with DFT-MD. The DFT-MD does not need to sample bond breaking and forming events. These will be added during RAL. We call the committee of bootstrap MLIPs “gen-0”. Subsequent RAL generations of MLIPs are denoted “gen-1”, “gen-2”, etc. 3. Automatically generate a set of products to use in constructing reactions to be used in RAL. In this work we used the Graph2SMILES model^42^ for the homogeneous phase reactions and CatKit: Catalysis Kit tool^43^ for the heterogeneous reactions. In practice, other methods could be used. 4. Automatically generate data needed to explore reaction paths with a given TS-finding method. Methods for doing this will be discussed for specific cases below. 5. Calculate reaction pathways and find TS using a randomly selected MLIP of the current generation in conjunction with a TS-finding tool. Filter the images produced and relabel with DFT. 6. The reaction pathways explored in the previous step will likely produce new molecules (stable intermediates and products) not contained in the initial training set. The configuration space of these new molecules should be trained with MD-AL. We used either self-consistent-AL (SC-AL) or nonself-consistent-AL (NSC-AL). Both of these approaches start by performing MLIP-MD on condensed phase systems containing all the molecules, both new and old, to generate filtered images to relabel with DFT and train a new committee of MLIPs. In the NSC-AL step we increment the RAL level, e.g., from gen-0 to gen-1, or gen-1 to gen-2, etc. In the SC-AL approach, we continue MLIP-MD-AL until the MLIPs converge to within a specified criterion (vide infra for details). Each cycle of MD-AL generates a new subgeneration of MLIPs, e.g., gen-1.1, gen-1.2, etc. We set a maximum of nine SC-AL subgenerations for each RAL generation to limit the amount of compute time devoted to SC-AL within a single RAL generation. Note that NSC-AL consists of running just one cycle of SC-AL. It is not clear a priori whether SC-AL or NSC-AL is better, since SC-AL is more rigorous but significantly more computationally demanding than NSC-AL. We compare the approaches for specific cases below. In addition to the SC-AL and NSC-AL approaches, we also explored a hybrid-AL approach, where the MLIPs were trained with a number of RAL generations with NSC-AL and then with SC-AL. In the specific case we examined (methanimine hydrolysis), this approach was used because standalone NSC-AL did not converge within a reasonable number of cycles. 7. Test for convergence. The MLIPs are considered converged when the images from reaction pathway sampling converge to within a specified criterion (vide infra for details). Otherwise, RAL continues by returning to step 5 to explore reaction pathways with the new generation of MLIPs.

We discuss each of the steps of the RAL workflow in more detail in the following paragraphs.

Step 1. Choosing the initial set of molecules to include in the bootstrap data set is usually dictated by the problem to be studied. In some cases one has only a set of initial reactants in mind and the goal is to identify as many meaningful reactions as possible. In this case, one would only include the reactants. If potential products, in addition to the reactants, can be proposed, then the initial set should include these products as well.

Step 2. Initial bootstrap training data were generated through short (around 10 ps) DFT-MD simulations at different conditions. Simulations in the *NVT* ensemble used the Nosé-Hoover^44^ thermostat. All DFT training data calculations were performed using the Vienna ab initio Simulation Package (VASP).^45–48^ Further details for the VASP simulations are provided in the Supporting Information. In practice, one could use the highest level of theory that is practical for the problem being studied. We varied thermostat temperature, density and composition in these simulations. We note that at the highest temperatures and densities, a few reactive events might be sampled in these DFT-MD simulations. In practice, sampling reaction events in the bootstrap training is beneficial, but not necessary. A committee of bootstrap MLIPs was trained using the same initial training data. Different seeds to the random initialization of MLIP training parameters result in different MLIPs that fit the PES in a nonuniform manner. We used a committee of four MLIPs in this work.

Steps 3–5. The bootstrap set of MLIPs was used to generate the first set of reaction pathways in the first round of RAL. There are three main components to this process: (1) product predictor, (2) TS exploration setup, and (3) MLIP-TS reaction exploration. These steps are shown schematically for homogeneous phase reaction sampling in Figure 1b. We used the Simplified Molecular Input Line Entry System (SMILES)^49^ representation for our reactants. SMILES representations of the products were generated using the Graph2SMILES model,^42^ which combines transformer models with molecular graph encoders to predict reaction outcomes. Other methods, such as Yet Another Reaction Program (YARP)^50^ could also be used for this step. The differences in the SMILES strings of reactants and products were used to automatically generate lists of bonds to be added and broken for each reactant–product pair. We made the assumption that an elementary reaction can consist of a maximum of four add and break commands (e.g., two add and two break, etc.). This is a practical limitation, which we enforce to eliminate reactions whose complexity can prevent the SE-GSM calculation from converging. If more than four add/break commands were required to produce the products, we decomposed these complex reactions into a series of elementary reactions that included reactions between intermediates to achieve the products predicted by Graph2SMILES. All sets of elementary add/break commands were used as input to the SE-GSM reaction exploration step, starting from the relaxed geometries of the reactants.

A comparable workflow for heterogeneous phase reactions is shown in Figure 1c, with several modifications to the algorithm relative to the approach for homogeneous systems. In the product predictor step, a reaction network was generated using the initial reactant using the CatKit tool.^43^ An enumerated list of products was generated based on the reactant’s SMILES representation and all possible geometries and topologies of each candidate product were generated using CatKit. Combinations of products were then formed by matching the stoichiometry of the products to the reactants. A catalytic surface was picked from a pool of surfaces as the candidate surface for reaction exploration. Each reactant and product pair was adsorbed onto copies of the surface and relaxed with a randomly selected MLIP to create the initial and final NEB images. The initial guesses for the NEB intermediate images were interpolated from these initial and final structures. We used five intermediate images (seven total) for our NEB calculations. This choice was guided by benchmarks on methane reaction examples used to validate our method, which showed that this number was sufficient to accurately capture the potential energy surface. Larger numbers of images can also be used without prohibitive cost, as the use of MLIPs makes NEB calculations inexpensive. We set index = −1 in CatKit, which returns all possible combinations of molecules adsorbed on different sites, increasing the total number of reaction paths to be explored. All NEB calculations were performed in the Atomistic Simulation Environment (ASE)^51^ using the “aseneb” method. The calculations employed a spring constant of *k* = 0.1 eV/Å and no climbing images were used. The FIRE^52^ optimizer was used, with a convergence criterion set to a maximum force threshold of 0.1 eV/Å and a cap of 500 NEB optimization steps. Limiting the maximum number of optimization steps also helped filter out poorly initiated geometries.

Step 6. We extracted stable intermediate structures from our explored set of reactions in order to train the MLIPs to account for these new species. This was done with MD-AL, which samples configurational space rather than chemical space, which is sampled by the reaction exploration part of RAL. Products from all converged reaction trajectories were considered candidates for potential stable intermediates. They were further filtered based on three criteria: (i) the reverse reaction barrier must be greater than 2*k*_B_ *T*, where *k*_B_ is the Boltzmann constant and *T* = 300 K is the temperature, (ii) relaxing the potential intermediate must not result in any bond rearrangements, (iii) the candidate must not be a radical, since radicals are assumed to be unstable; this was determined by empirically calculating the multiplicity of the relaxed product. Candidates that pass these criteria were populated in a cubic box, 20 Å on a side, with other stable intermediates and reactants using PACKMOL^53^ at liquid-like densities. These structures were used as initial geometries for *NVT* MLIP-MD simulations at temperatures ranging from 600 to 1000 K in our SC-AL or NSC-AL training.

Step 7. These explored configurations were filtered with the committee of MLIPs based on the maximum force deviation, which is an uncertainty metric used in DP-GEN^8^ defined as

1 $$\epsilon =\max_{i}\left[\sqrt{\left\langle {∥F_{\omega ,i}\left(R\right)-\left\langle F_{\omega ,i}\left(R\right)\right\rangle ∥}^{2}\right\rangle }\right]$$

where $F_{\omega ,i}\left(R\right)$ is the force on atom *i* in a given configuration R , ω denotes the set of parameters in the MLIP, and the angle brackets ⟨···⟩ denotes the expectation values with respect to the MLIP committee members. Configurations were classified as good, candidate, and rejected based on whether their maximum force deviation fell below, between, or above predefined lower and upper threshold values. This classification approach is similar to the filtering mechanism used by DP-GEN.^8^ For all systems, we set our upper threshold to 1 eV/Å and our lower threshold to 0.1 eV/Å, although we sometimes relaxed the upper limit to included values as high as 2 eV/Å, if a simulation resulted in very few filtered images. Filtered images (falling in the candidate range) were then relabeled with DFT. We only included configurations and corresponding DFT energies and forces in our training database if the following criteria were satisfied: (i) the total energy of the system must be less than the atomization energy, (ii) the total magnetic moment must be an integer (to within a tolerance of 10^–3^ μ_B_), (iii) the electronic self-consistent field calculation must converge within 200 steps. The SC-AL cycle was considered converged if ϵ values for at least 95% of the configurations explored in the last MD-AL cycle fell below 0.5 eV/Å for a given subgeneration. The RAL process was considered converged when the ϵ values for at least 90% of the configurations explored in the reaction paths fell below 0.5 eV/Å for a given generation.

In addition to the maximum force deviation convergence metric, the MLIPs were also evaluated based on the uniform manifold approximation and projection (UMAP)^54^ convex hull volume and cumulative nearest neighbor (NN) distance metrics. These evaluation metrics are discussed in detail in the Supporting Information. In summary, the convex hull volume is an indicator of the diversity of structures explored in each RAL cycle. The larger the volume, the more diverse the exploration. The cumulative NN distance metric is a measure of the diversity of the structures explored in generation *n* compared with all the structures explored in the previous *n* – 1 generations. A significant decrease in this value indicates that we have already explored most of the available structures.

### 2.2 Reaction Systems

We here discuss details of implementing RAL to each of the three systems we studied.

#### 2.2.1 Uncatalyzed Ammonia Synthesis Reaction

We chose to study the gas-phase ammonia synthesis reaction due to its simplicity. The reaction involves only two elemental molecules, nitrogen and hydrogen, reducing the complexity of the chemical space. Additionally, it presents numerous possible intermediates and avoids complications arising from multiple spin states, which are encountered in reactions involving molecules like O_2_. We note that our MLIP is not capable of accounting for spin states, so there is no way to distinguish between singlet and triplet O_2_, for example. The high reaction barrier associated with breaking the N≡N bond further adds to the challenge, making it an interesting candidate for developing and evaluating our RAL algorithm.

The initial bootstrap training data were generated from 11 *NVT* DFT-MD simulations, each about 10 ps long, of periodic simulation cells containing H_2_, NH_3_, and N_2_. The density of the system and the thermostat temperature (1000–7000 K) were varied. We collected images 25 fs apart from these simulations (to avoid highly correlated configurations) and relabeled them at the same settings but with spin-polarization. Additional computational details on the DFT, MLIPs and TS-finding methods are mentioned in the Supporting Information.

SMILES representations of N_2_ and H_2_ reactants were used as input to Graph2SMILES and we selected the top six products from the list of predicted products. These products are (1) NH_3_, (2) N_2_H_4_, (3) N_2_H_2_, (4) N_2_H_2_^2+^, (5) N_2_H_3_^+^, and (6) H. The SMILES representation of reactants and products are listed in Table S1. Reactions corresponding to products 3, 4, and 6 are elementary reactions. All other reactions are multistep. We generated a total of 99 SE-GSM reactions for these six products. Several stable intermediates were discovered from our MLIP-based SE-GSM. Rejected configurations above the 1 eV/Å threshold typically were configurations with high energy or were radicals. We trained this system with both the NSC-AL and SC-AL approaches.

After the MLIPs were converged, we tested the accuracy and completeness of the training by comparing the PES computed from our MLIPs with DFT values for a set of benchmark reaction mechanisms reported in the literature.^55^ This comparison was a predictive test of the MLIP because the mechanisms from the literature were not included in our RAL training. Note that this comparison with benchmark reactions is not part of the RAL formalism, since we assume that in many cases one will not have a priori knowledge of the reaction products and will therefore not have access to benchmark reactions. We performed benchmark testing here as a proof that RAL is capable of predicting the PES for reactions not in the training set with reasonable accuracy. A set of four gas-phase elementary reactions for ammonia synthesis were taken from Hwang and Mebel.^55^ They calculated a series of different mechanisms at the MP2/6–31G** level of theory, using the intrinsic reaction coordinate (IRC)^56^ method to find minimum energy paths from transition state structures to products and reactants. The reaction mechanism we selected is just one of several possible mechanisms reported in Figure 1 of Hwang and Mebel.^55^ It is not reasonable to compare reaction geometries and energies computed with MP2 to our MLIPs, which were trained at the PBE/D3 level of theory. Therefore, we used the mechanisms from Hwang and Mebel as starting points for our own NEB calculations in VASP.

#### 2.2.2 Methanimine Hydrolysis Reaction

The methanimine hydrolysis reaction consists of two reactants, CH_2_NH and H_2_O, which can form a variety of small molecule products. A bootstrap MLIP was trained with ten *NVT* DFT-MD simulations of periodic simulation cells consisting of CH_2_NH and H_2_O at liquid-like densities at 1000 K.

We used Graph2SMILES^42^ with CH_2_NH and H_2_O as the set of reactants and chose the following five products: (1) CH_3_NH_2_, (2) CH_2_NH_2_OH, (3) CH_2_O, (4) H_2_, and (5) CH_3_NHOH. The SMILES representations for reactants and products are listed in Table S1. Reactions corresponding to products 2 and 4 are elementary reactions, and the rest are multistep reactions. We generated a total of 170 SE-GSM reactions for these five products.

We used the hybrid-AL and SC-AL approaches to train two sets of MLIPs for this system. We initially attempted to use the NSC-AL approach, but we noticed that RAL generations 3–8 (gen-3 to gen-8) showed only small improvements in the maximum force deviation metric. We therefore adopted a hybrid approach: eight generations of MLIPs were trained with the NSC-AL cycles followed by two generations with SC-AL, at which point the desired convergence was reached. For the SC-AL approach, the MLIPs converged after three generations of RAL.

After the MLIPs were converged, we tested their accuracy by comparing with a benchmark hydrolysis mechanism reported by Ali,^57^ who used IRC calculations carried out at the M06–2*X*/6–311++G­(3df,3pd) level of theory to identify a mechanism involving three transition states to form CH_2_O and NH_3_. We recalculated the reaction pathways with VASP-NEB to allow for a meaningful comparison with the MLIP energies. We note that our RAL pathways did not include the pathway identified by Ali; indeed, the products we selected from Graph2SMILES did not include NH_3_.

#### 2.2.3 Methane Activation and C–C Coupling Surface Catalysis Reaction on Titanium Carbide

We used the RAL scheme depicted in Figure 1a,c to train MLIPs for the methane activation and coupling reaction on titanium carbide (Ti_*x*_C_*y*_) surfaces. The conversion of methane into value-added products without generating CO_2_ is a critical challenge for the sustainable chemical industry. Methane lacks low-energy empty orbitals, high-energy filled orbitals, and a permanent dipole or quadrupole moment, making it difficult to activate and engage in chemical reactions. This intrinsic stability makes methane activation a stringent test for any reactive learning method. Furthermore, most conventional catalysts are unsuitable for this transformation, whereas transition metal carbides, such as titanium carbide, have been computationally demonstrated to be effective for methane activation.^58,59^

Initial structures for Ti_*x*_C_*y*_ were sourced from the Materials Project,^60^ where we selected Ti_2_C, TiC, and Ti_8_C_5_ as stable structures. After relaxing these bulk structures, we performed high-temperature (up to *T* = 2500 K) *NVT* DFT-MD simulations. Each simulation ran for 20 ps with a 1 fs time step, and configurations were sampled every 80 fs for our initial training data. Surface geometries were generated by cleaving the relaxed bulk structures using CatKit^43^ along the (100), (110), and (111) planes. The same DFT-MD protocol was used to generate training data for these surface geometries. Additionally, two systems were modeled to capture the dynamics of methane and ethane molecules. In the first system, methane and ethane molecules at liquid-like densities were simulated at *T* = 2000 K for 10 ps using a 0.25 fs time step, while in the second, methane and ethane molecules were placed on both sides of the (100) surface of Ti_2_C, with a simulation time of 5 ps at *T* = 1000 K and a 0.5 fs time step. These configurations were used to generate our initial set of MLIPs. We then conducted two iterations of MD-AL to obtain our initial bootstrap MLIP. We used the starting structures from our initial DFT-MD data generation step as our initial geometries for MD-AL. All simulations were run for 0.5 ns at *T* = 1000 K.

We trained our MLIPs for Ti_*x*_C_*y*_ reactions exclusively using a simplified NSC-AL scheme, only performing a single MD-AL cycle using the original system, rather than including new intermediates from each RAL cycle. Only three generations of RAL MLIPs were required to achieve convergence. Each generation contained two data generation steps: NEB and MD at 2000 K for AL. The process of using NEB to generate new data is shown in Figure 1c. We picked three different initial structures to explore for each generation. Ti_2_C was used for the first generation, TiC for the second and Ti_8_C_5_ for the third. The (100) surface was used for all structures. We treated ethane as the reactant and performed an exploration of ethane decomposition products. Initial structures for the reactant were generated by placing ethane molecules on all possible sites of the surface. There were four surfaces in the first generation, 12 in the second, and 13 in the third, which were then geometrically relaxed using MLIPs from the previous generation. There were 18 total intermediates (composed of C and H atoms) that were used to make a pool of 43 possible products. The initial adsorption site for each molecule in a given product combination was chosen randomly. Each of the products were further relaxed using a randomly picked MLIP from the previous generation. There were 43 MLIP-NEB simulations for each reactant in a given generation, which gave 172 reactions for the first generation, 516 for the second, and 559 for the third, which totals to 1247 reactions.

The final MLIPs from the RAL process were tested against benchmark DFT calculations for C–C coupling on the Ti_2_C surface. We chose the benchmark reactions from reactions reported by Kuriakose et al.^61^ on the Ti_2_C­(100) surface. Their reaction geometries were generated with Quantum ESPRESSO.^62^ We used reaction pathway coordinates kindly provided by Prasenjit Ghosh for CH_3_–CH_3_, CH_2_–CH_2_, and CH–CH coupling as initial guesses for our own NEB calculations using VASP. The CH_3_–CH_3_ coupling reaction pathway consists of three elementary reactions involving (1) CH_3_–CH_2_ bond formation, (2) H hopping, and (3) CH_2_–H bond formation. The CH_2_–CH_2_ coupling reaction pathway involves two elementary reaction steps: (1) CH_2_ hopping and (2) CH_2_–CH_2_ bond formation. The CH–CH coupling reaction pathway is elementary. Thus, our benchmark reactions consist of a total of six NEB pathways. None of these reaction pathways were included in our training calculations.

We used the final set of converged MLIPs to study the catalytic reactivity of TiC, Ti_2_C, and Ti_8_C_5_ surfaces using MD. Simulations were performed at different temperatures. We constructed 4 × 4 × 1 supercells, containing about 900 atoms. Studying surface reactivity at such large scales is infeasible with conventional ab initio methods. The (001) and (100) surfaces were chosen for TiC and Ti_2_C, respectively. Stable surfaces for Ti_8_C_5_ have not been reported in the literature. We calculated surface energies for the (100) and (001) surfaces and found that the latter has a lower surface energy; we therefore used the (001) surface in our calculations. All surfaces had vacuum spacing of 10 Å, making slit pore geometries. We conducted two types of simulations to assess the reactivity of these surfaces. The first set included running *NVT*-MD simulations at 1200 K, starting with 20 CH_4_ molecules initially randomly placed in the void space for each of these three systems for 500 ps. We extended the MD simulation for Ti_2_C system to a total of 1.5 ns, focusing on methane decomposition and surface intermediates on the surface. Ten independent simulations were performed for all systems to improve statistical accuracy. The second set consisted of short 10 ps simulations to calculate initial CH_4_ decomposition rates on Ti_2_C and Ti_8_C_5_. As a part of the first set, we performed simulations at three dosing stages in Ti_2_C. We added 20 CH_4_ molecules in the simulation cell as an initial dose and ran the simulation. At the end of the first simulation, we deposited 20 additional CH_4_ molecules. Another 20 molecules were added in the third dose. Methane molecules from each dose reacted with the surface sites of Ti_2_C and provided information about the surface activity for CH_4_ decomposition. The simulations were run for 500 ps each with 0.5 fs timesteps, collecting trajectory data after every 100 timesteps. The trajectory data were used to calculate intermediates formed during the simulation using the Python NetworkX library. The number of intermediates was quantified by counting the number of atoms attached to the C atoms in CH_4_ during the simulation. Initial rate calculations for the Ti_2_C and Ti_8_C_5_ systems were performed by running 10 ps long simulations for four different temperatures, starting with 20 CH_4_ molecules. Trajectories were collected every 10 timesteps. The rate was calculated by determining the slope of the initial trend, which was fitted to a line using data from the initial linear range in the plot. We ran 100 independent runs to improve the statistics for the number of CH_4_ molecules for a given time step.

## 3 Results and Discussion

### 3.1 Uncatalyzed Ammonia Synthesis Reaction

We developed two sets of MLIPs for the uncatalyzed ammonia synthesis reaction using the NSC-AL and SC-AL approaches. The results for the converged MLIPs using the NSC-AL approach are shown in Figure 2. The MLIPs converged after four generations of RAL, as shown in Figure 2a. The percentage of SE-GSM images with ϵ < 0.5 eV/Å increased with each successive generation of RAL. Specifically, 91.15% (1153 images; see Figure S1) of SE-GSM images have ϵ < 0.5 for the gen-4 MLIP. The gen-4 kernel density plot has a single maximum close to ϵ ∼ 0, indicating that the majority of SE-GSM images have very low ϵ values. In contrast, the kernel density distribution of gen-2 MLIPs exhibits two main peaks, suggesting greater deviations in ϵ values compared to gen-4 MLIPs. For the gen-2 MLIPs, 79.45% (1005 images) have ϵ < 0.5 eV/Å. Finally, the gen-0 MLIPs, which is the bootstrap model, shows multiple peaks in its kernel density plot, indicating even higher deviations on average. Only 65.71% (205 images) have ϵ < 0.5 eV/Å for gen-0 MLIPs. These results suggest that with increasing generations of RAL, the MLIPs become progressively more accurate and more certain.

The convex hull volumes for four generations of MLIPs with the NSC-AL approach are shown in Figure 2b. The overlapping and individual UMAP plots for each generation are shown in Figure S2. The volume increases with each new generation of RAL, indicating that a larger configuration and chemical space was explored with each subsequent generation of RAL. To understand whether we are sampling distinct configurations, we calculated the cumulative NN distance as shown in Figure 2c. The cumulative NN distance decreases with each generation of RAL, suggesting a reduction in the number of distinct configurations sampled. This indicates that the RAL is approaching complete sampling of the reactions for the chosen products.

**Figure 2:**
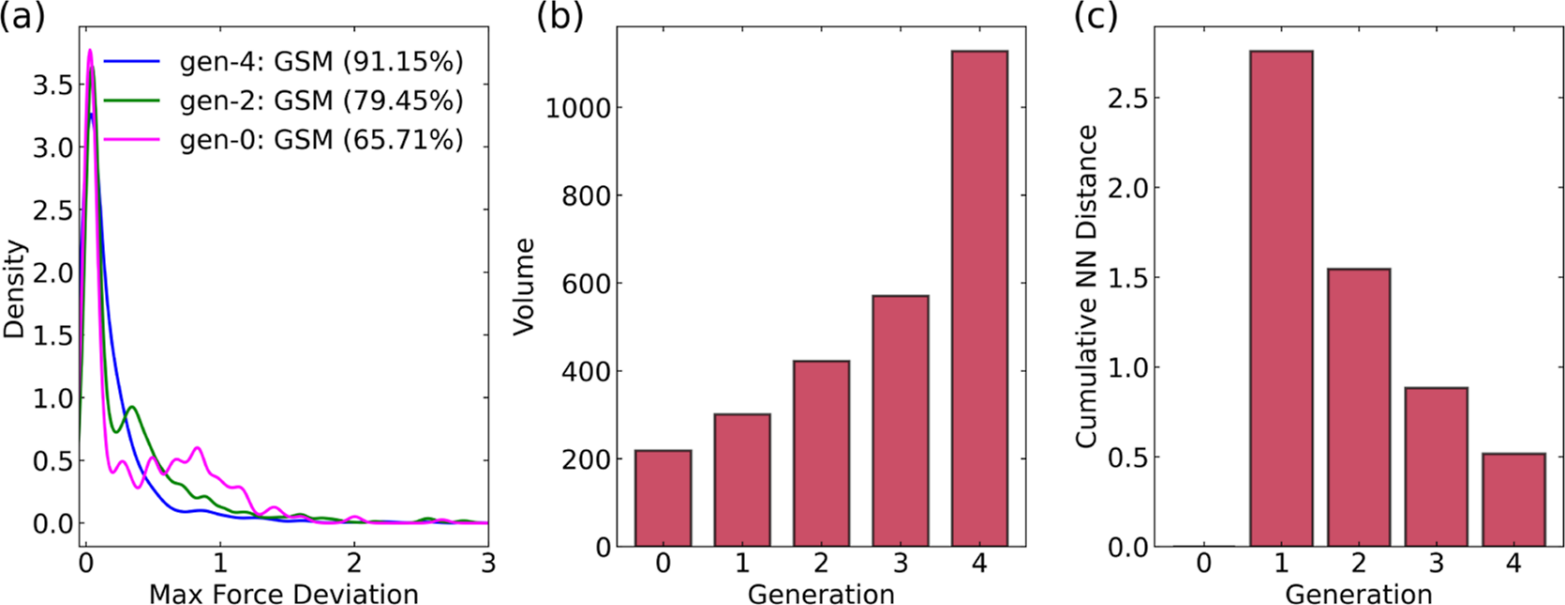
(a) Maximum force deviations for SE-GSM images for the uncatalyzed ammonia synthesis system with the NSC-AL approach for RAL gen-0, gen-2, and gen-4. Other generations are not shown for clarity. The percentage of images with maximum force deviations less than 0.5 eV/Å is given in parentheses. (b) Convex hull volumes for RAL generations gen-0 to gen-4. (c) Cumulative NN distance between consecutive generations from gen-0 to gen-4.

The SC-AL MLIPs converged after five generations of RAL, as shown in Figure 3a. Similar to the NSC-AL approach, we found that the kernel density plot for the converged generation (gen-5.1) has a single main peak near ϵ = 0, with 91.66% (1330 images; see Figure S3) of the SE-GSM images having ϵ < 0.5 eV/Å. Gen-0, gen-1.6, and gen-3.7 have 59.45% (151 images), 78.72% (1143 images), and 86.03% (1250 images) of SE-GSM images with ϵ < 0.5 eV/Å, respectively. The convergence of the configuration space, i.e., the SC-AL convergence, is shown in Figure 3b. The initial bootstrap MLIP has essentially no MLIP-MD images with ϵ < 0.5 eV/Å. SC-AL converges for gen-1, 3, and 5 in six, seven, and one AL cycles, respectively. Gen-1.6, gen-3.7, and gen-5.1 have 95.69%, 96.75%, and 96.88% (174402 images) of MLIP-MD images with ϵ < 0.5 eV/Å. We again note that the SC-AL generation notation (e.g., gen-1.6) indicates that 6 AL-MD cycles were performed before achieving convergence for generation 1.

**Figure 3:**
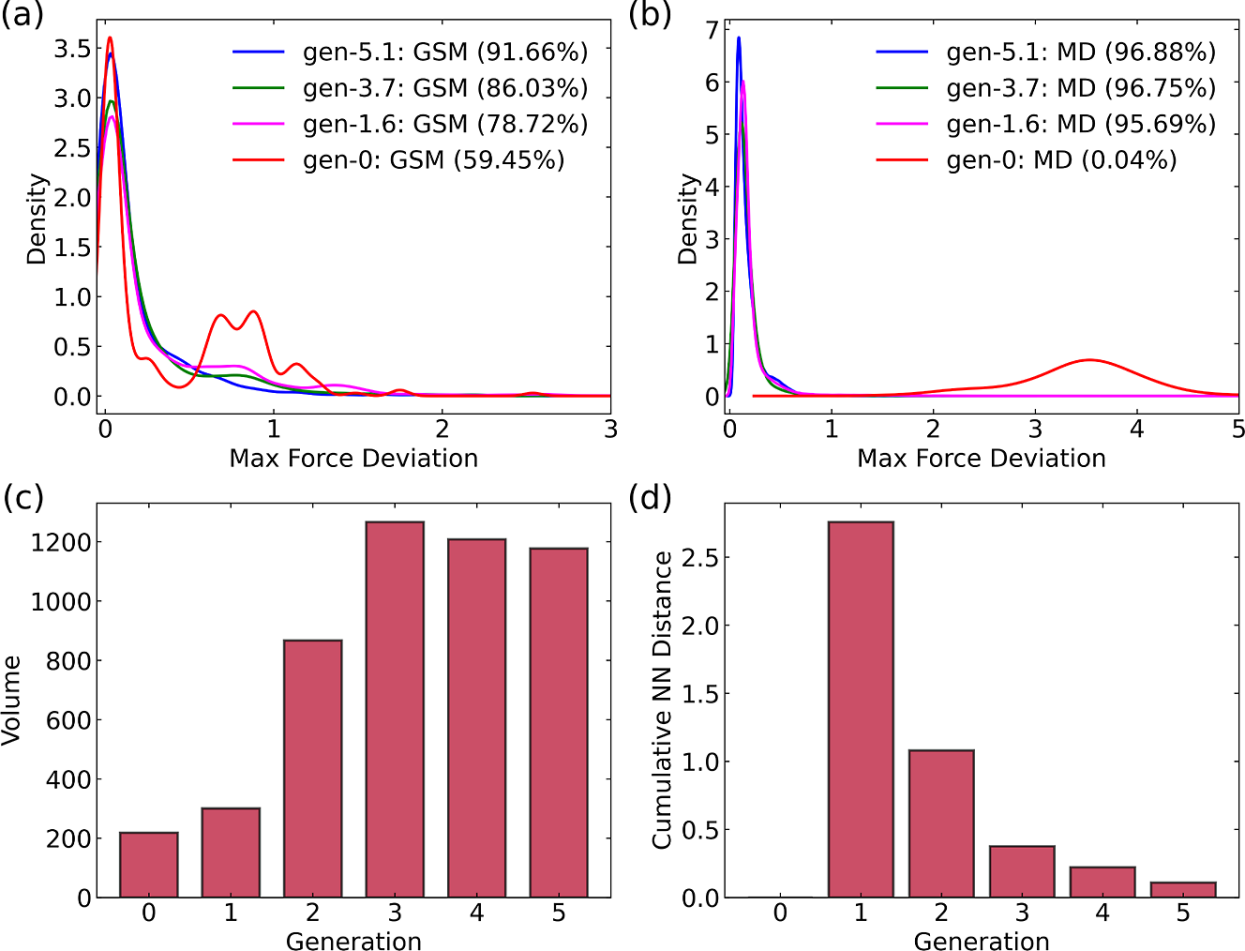
Maximum force deviations for (a) SE-GSM and (b) MD images for the uncatalyzed gas-phase ammonia system with the SC-AL approach for RAL gen-0, gen-1.6, gen-3.7, and gen-5.1. Other generations are not shown for clarity. The percentage of images with maximum force deviations less than 0.5 eV/Å is given in parentheses. (c) Convex hull volumes for gen-0 to gen-5 RAL generations. (d) Cumulative NN distance between consecutive generations from gen-0 to gen-5.

In Figure 3c, the convex hull volume increases from gen-0 to gen-3 and remains large for gen-4 and gen-5 MLIPs. The UMAP plots of gen-0 to gen-5 MLIPs with the SC-AL approach are shown in Figure S4. The cumulative NN distance plot is shown in Figure 3d. The cumulative NN distance decreases with each generation of RAL. By gen-5, the cumulative NN distance has a very small value, indicating that RAL is less effective at generating truly new structures. This implies that RAL has already sampled most of the configurational and chemical space available to it for the given products. The sampling of new chemical space might still be possible if different products were generated from Graph2SMILES. We note that all convex hull volumes are reported per generation, and thus their trends do not necessarily indicate convergence—unlike the NN distance metric, which is cumulative. For instance, gen-4 of NSC-AL in Figure 2b,c shows a relatively large convex hull volume but the smallest NN distance, suggesting that the sampled configurations, while diverse, largely overlap with previously explored regions. We also note that the final cumulative NN distance for the SC-AL (0.1) is significantly smaller than the NSC-AL (0.5), as seen by comparing Figures 2c and 3d. This indicates that the SC-AL approach is closer to exploring all possible configurations than the NSC-AL approach for each of the terminal MLIPs.

The benchmark reaction PES calculated from DFT for the four reaction pathways taken from Hwang and Mebel^55^ are shown in Figure 4a-d in black. The predicted reaction PES trained with RAL using the NSC-AL gen-4 and SC-AL gen-5.1 MLIPs are shown in red and blue, respectively. Error bars, based on the range between the lowest and highest energy predictions among the ensemble of four MLIPs, are included and are small for all four reactions, indicating strong agreement among the models in energy prediction. The mean absolute errors (MAEs) for the reaction energy pathways, as well as the forward and reverse barriers predicted by both models, are given in Table 1. We note that the errors in the energy barriers are based on relative energy differences, not on the values of the actual energies. Hence, offsets in the MLIP and DFT energies will cancel out. On average, the MLIPs trained with SC-AL (SC-RAL) gen-5.1 MLIP yields lower MAEs than the MLIPs trained with NSC-AL (NSC-RAL) gen-4 MLIP, except for reaction 1. On average, the prediction errors for reaction PES remain below 0.5 eV for all reactions except for the third reaction.

**Figure 4:**
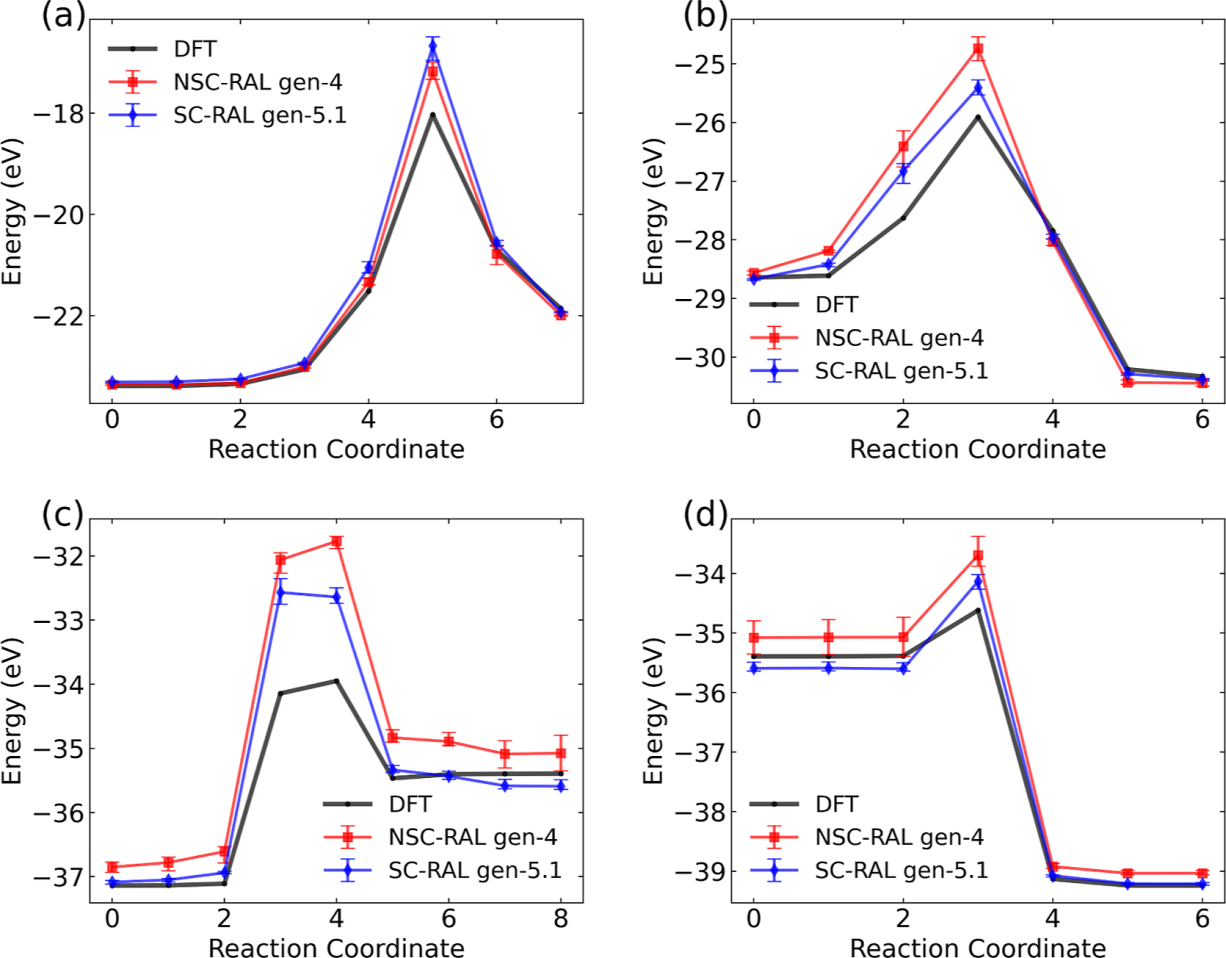
(a-d) Uncatalyzed gas-phase ammonia synthesis benchmarking reactions computed from SC-RAL gen-5.1 (blue, diamonds) and NSC-RAL gen-4 (red, squares) compared against DFT (black, circles). The error bars represent the largest and smallest predicted energies across four MLIPs. The reactions are (a) N_2_ + H_2_ → cis– HNNH (b) cis– HNNH + H_2_ → H_2_NNH_2_ (c) H_2_NNH_2_ + H_2_ → HNNH_3_ + H_2_ (d) HNNH_3_+H_2_ → NH_3_+NH_3_.

**Table 1:** Uncatalyzed Gas-phase Ammonia Synthesis Mean Absolute Error (MAE) for the reaction PES and Forward and Reverse Barrier Predictions, in Parentheses, with the NSC-RAL Gen-4 and SC-RAL Gen-5.1 MLIPs^a^

model	NSC-RAL	SC-RAL
reaction 1	0.17 (0.83, 0.99)	0.30 (1.28, 1.43)
reaction 2	0.49 (1.09, 1.29)	0.25 (0.52, 0.54)
reaction 3	0.79 (1.89, 1.86)	0.41 (1.35, 1.60)
reaction 4	0.36 (0.61, 0.72)	0.17 (0.69, 0.46)

a The energies are reported in eV.

Both NSC-RAL and SC-RAL MLIPs show significant errors in predicting the forward and reverse barriers for reaction 3, indicating poor performance in estimating the transition state energy. This indicates that our RAL sampling did not explore configurations that were close to the TS structure for reaction 3. This reaction is a rather unusual hydrogen exchange reaction (see Figure 4c). In fact, none of the SE-GSM reactions involved the stoichiometry for that reaction, having 6 H atoms and 2 N atoms, so reaction 3 is an extrapolation well outside the training of the MLIP.

The reaction PES and associated uncertainty estimates for each reaction, predicted using NSC-RAL MLIPs (gen-0 to gen-4) and SC-RAL MLIPs (gen-0, gen-1.6, gen-2.4, gen-3.7, gen-4.5, and gen-5.1) are presented in Figures S5 and S6. We estimated these uncertainties by computing the mean standard deviation along the reaction coordinate for each reaction. The uncertainty decreases with increasing RAL generations for both approaches.

### 3.2 Methanimine Hydrolysis Reaction

We developed two sets of MLIPs for the methanimine hydrolysis reaction using the hybrid-AL and SC-AL approaches. The hybrid-AL MLIP was trained with NSC-AL from gen-0 to gen-8 and then with SC-AL for gen-9 and gen-10. The final MLIP converged in gen-10.2. The kernel density plot for SE-GSM images for gen-0 to gen-10.2 is shown in Figure S7. The percentage of SE-GSM images with ϵ < 0.5 eV/Å increased slowly from gen-0 to gen-8. Adding SC-AL training converged the MLIPs faster. The results for the converged MLIP using the hybrid-AL approach are shown in Figure S8. 90.17% of the SE-GSM images (486 images, see Figure S9) and 97.11% of the MLIP-MD images (58268 images) have ϵ < 0.5 eV/Å. The convex hull volume was large for most generations of RAL (Figure S8c) and cumulative NN distance decreased with increasing generations of RAL (Figure S8d), indicating RAL is approaching complete exploration of the reaction space defined by the five products we selected from Graph2SMILES. The overlapping and individual UMAP plots for the hybrid-AL approach are shown in Figure S10.

We also trained MLIPs with the SC-AL approach for the methanimine hydrolysis system as we did for the ammonia system. Our SC-AL MLIPs converged at gen-3.9. We note that the SC-AL approach converges faster than the hybrid approach, again indicating that SC-AL is a more effective approach to training MLIPs than the NSC-AL approach or the hybrid-AL approach. The results of converged MLIPs with the SC-AL approach are shown in Figure S11. 93.42% (1775 images; Figure S12) and 95.29% (409770 images) of SE-GSM and MLIP-MD images have ϵ < 0.5 eV/Å. We note that the SC-AL approach yields more SE-GSM intermediates that have ϵ < 0.5 eV/Å than the hybrid approach, resulting in a larger exploration of the chemical space with this approach. This is also true for exploring the configurational space. The SC-AL approach yields a larger number of MLIP-MD images with ϵ < 0.5 eV/Å than the hybrid approach. The convex hull volume increases with increasing generation of RAL and the cumulative NN distance decreases with increasing generation of RAL. The overlapping and individual UMAP plots are shown in Figure S13. While the convex hull volume is similar for the converged MLIP with the hybrid-AL and SC-AL approach, the cumulative NN distance of the hybrid-AL approach is significantly smaller than the SC-AL approach (see Figures S8d and S11d). This indicates that while our SC-AL MLIP converged in gen-3.9, the chemical and configurational space should probably be explored further. One could add a maximum cumulative NN distance as an additional convergence criterion to ensure more complete exploration.

We assessed the performance of the converged hybrid-AL and SC-AL MLIPs by comparing MLIP calculations of the reaction PES and forward and reverse barriers for reaction pathways identified by Ali^57^ with DFT. To ensure consistency in the level of theory, all benchmark reactions were recomputed using NEB calculations at the same DFT level of theory as used to train the MLIPs. The comparison is shown in Figure 5. The MAE for the reaction PES and forward and reverse barrier predictions are summarized in Table 2. The MAE for predicting the reaction PES of reaction 1 is lower with the SC-AL gen-3.9 than the hybrid gen-10.2 MLIP. However, hybrid gen-10.2 MLIP predicts forward and reverse barriers slightly better than the SC-AL gen-3.9 MLIP. Both MLIPs predict similar reaction PES as well as forward and reverse barriers for reaction 2. Although the absolute error is small for this reaction, the reaction barrier itself is very low, which may result in large a percentage error. For reaction 3, hybrid gen-10.9 MLIP predicts lower MAE than SC-AL gen-3.9. Overall, the performance of both of these MLIPs is similar. The reaction PES and associated uncertainty estimates for each reaction, predicted using hybrid MLIPs (gen-0, gen-2, gen-4, gen-8, and gen-10.2) and SC-AL MLIPs (gen-0, gen-1.9, gen-2.7, and gen-3.9) are shown in Figures S14 and S15. The accuracy of MLIPs in predicting the reaction PES increases with increasing generations of RAL.

**Figure 5:**
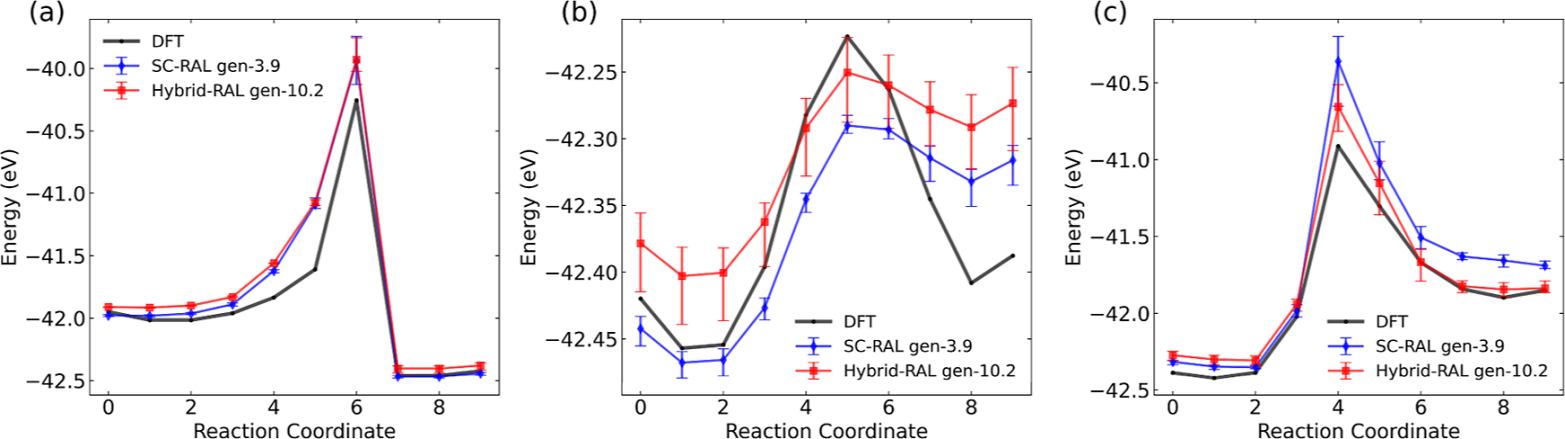
Methanimine-water benchmarking reactions for MLIPs trained with SC-RAL gen-3.9 (blue, diamonds) and hybrid-RAL gen-10.2 (red, squares) compared against DFT (black, circles) in eV. The error bars represent the largest and smallest predicted energies across four MLIPs. The reactions are (a) CH_2_NH + H_2_O → NH_2_CH_2_OH (b) NH_2_CH_2_OH → NH_2_CH_2_OH (c) NH_2_CH_2_OH → CH_2_O + NH_3_·.

**Table 2:** Methanimine-Water System Mean Absolute Error (MAE) for the reaction PES and Forward and Reverse Barrier Predictions, in Parentheses, for MLIPs Trained with SC-RAL Gen-3.9 and Hybrid-AL Gen-10.2^a^

model	SC-RAL	hybrid-RAL
reaction 1	0.13 (0.34, 0.33)	0.17 (0.29, 0.28)
reaction 2	0.04 (0.04, 0.14)	0.05 (0.07, 0.14)
reaction 3	0.18 (0.48, 0.39)	0.09 (0.14, 0.24)

a The energies are reported in eV.

### 3.3 Comparison of RAL with the Nanoreactor Method

In order to assess the efficiency of the RAL formalism, we have compared the performance of RAL with a state-of-the-art training method from the literature. We chose to compare RAL with the Nanoreactor active learning (NR-AL) approach developed by Zhang et al.^20^ because like RAL, NR does not require a priori knowledge of reaction pathways. NR-AL is an MLIP AL method that uses long MLIP-MD trajectories having extreme fluctuations in temperature and volume to sample reaction events. We trained MLIPs for the methanimine system using the NR-AL method and compared the computational cost and accuracy of predicting the benchmark reactions with our RAL MLIPs.

We implemented the NR-AL approach as described in the Supporting Information. Briefly, we started with the same bootstrap gen-0 MLIPs used above for starting RAL training. We trained a total of 47 generations of MLIPs with the NR-AL method. This number of generations effectively used about the same amount of computing resources as the RAL approach, as measured in service units (SUs). See the Supporting Information for details. Energies for the benchmark reactions from RAL and NR-AL for the methanimine system are shown in Figure 6. We found that NR-AL had not converged within 47 generations, as demonstrated by the large error bars, which indicate poor agreement between the committee of MLIPs trained with NR-AL. We also observe that the NR-AL MLIPs are unable to accurately reproduce the PES of the reactions, exhibiting offsets of several eV from the DFT energies in some cases. Moreover, the NR-AL MLIPs only gave an actual reaction barrier for reaction (a) in Figure 6. The other two PESs calculated from the NR-AL MLIPs show monotonic increases in energy. The forward and reverse reaction barriers, which do not depend on the values of the energies, but only on relative differences, are reported in Table 3, along with the mean absolute errors in the potential energy surfaces. For reactions (b) and (c) in Figure 6 we only report forward barriers, since the monotonically increasing PESs give negative reverse barriers.

**Figure 6:**
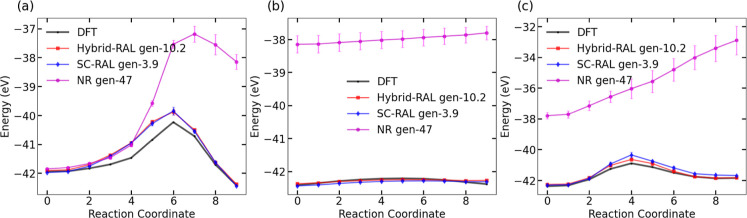
Predicted energy profiles along the reaction coordinate for the three benchmark reactions in the methanimine-water system. Results are shown for DFT (black, small circles), hybrid-AL gen-10.2 (red, squares), self-consistent-AL (SC-AL) gen-3.9 (blue, diamonds), and NR-AL gen-47 (magenta, circles). The reactions are (a) CH_2_NH + H_2_O → NH_2_CH_2_OH (b) NH_2_CH_2_OH → NH_2_CH_2_OH (c) NH_2_CH_2_OH → CH_2_O + NH_3_.

**Table 3:** Mean Absolute Error (MAE) for the reaction Energy Pathway, with Errors in the Forward and Reverse Barriers (in Parentheses) Using the Methanimine-Water NR-AL Gen-47 MLIP^a^

model	NR-AL (eV)
reaction 1	1.70 (2.96, 1.22)
reaction 2	4.30 (0.14, −)
reaction 3	6.10 (3.41, −)

a Reverse barriers are not reported for reactions 2 and 3 because the PES is monotonically increasing (see Figure 6). All results are reported in eV.

We note that Zhang et al.^20^ stated that the NR-AL method may not perform well for gas-phase reactions. We suspect that the poor PES performance of NR-AL for the benchmark reactions is because each NR-AL generation explores only a small fraction of the relevant chemical and configurational space. This is evident from the UMAP plots of intermediates generated with selected NR-AL generations, shown in Figure S16. A visual comparison of the UMAP plots from RAL (Figures S10 and S13 for methanimine) with the corresponding NR-AL plots provides evidence that RAL explores much greater volumes of chemical space in each generation. Moreover, the density of points generated from NR-AL is much lower than for RAL. We computed the convex hull volumes and cumulative NN distances for each NR-AL generation using our UMAP information. These are shown in Figure S17. The convex hull volumes of the NR-AL generations of the methanimine system were all about 2 orders of magnitude smaller than the gen-0 volume, indicating low diversity in the generated intermediates compared to bootstrap training. Due to the sparse distribution of points in NR-AL, the cumulative NN distance values are larger than RAL. Moreover, there is no decrease in the values over the generations, indicating a lack of convergence in exploring chemical space. Both RAL and NR-AL started the AL from the same bootstrap potential, which performed poorly in predicting reaction pathways. However, RAL converged to correct reaction pathways after 3 generations of SC-AL and 10 generations of hybrid RAL for the methanimine-water system (Figures S14 and S15), while the NR-AL approach failed to converge to the DFT energies, as shown in Figure 6. The computational costs of the RAL and NR-AL training for each system are given in Table S3. We have broken out the SUs used for training, exploration, and relabeling. For methanimine, the SC-AL method used 67168 SUs, and the hybrid approach required 92944 SUs in total. We estimate that the effective computational cost required to reach NR-AL gen-47 is about 89000 SUs. Running NR-AL to convergence would, of course, require more SUs.

### 3.4 Methane Activation and C–C Coupling Surface Catalysis Reaction on Titanium Carbide

The converged MLIP results for the TiC systems are shown in Figure S18. As shown in Figure S18a, the percent of NEB images with ϵ < 0.5 increases with increasing generations of RAL. Unlike the case of ammonia and methanimine, the increase in percentage is very rapid, going from 1.27% to 90.55% in gen-1 to gen-2, respectively. The reason for such a drastic improvement from gen-1 to gen-2 can be attributed to the lack of reactive data to train the first generation, before RAL was performed. The convergence of MD-AL is reported in Figure S18b. The percentage of images with ϵ < 0.5 is greater than 95% for all generations. The volume of the convex hull is reported in Figure S18c. We see a decrease going from gen-0 to gen-1. This is because of the very poor quality of gen-0 for accounting for reactions, which resulted in almost all of the NEB images having larger maximum force deviations than the upper limit, resulting in very few NEB images being filtered for gen-1. Plots of data points in UMAP space and their convex hulls for each generation are shown in Figure S19. The volumes of gen-2 to gen-4 are acceptably large. The cumulative NN distances are shown in Figure S18d. We see that the distance does not decrease with each generation, as observed for the previous two systems. We do not expect this distance to decrease as we use different systems (surfaces) for each generation. In hindsight, we believe it would have been better to include all surfaces and to carry out MD-AL with all NEB structures explored in each generation. Nevertheless, the approach we used here as a proof-of-concept was accurate enough for our purposes.

The performance of RAL gen-4 with respect to the DFT benchmarking reactions is shown in Figure 7. The reaction PES computed from RAL agrees well with the DFT PES. We observe a systematic improvement in prediction quality with each generation of RAL, shown in Figure S20a.1–f.1. We also see a reduction in prediction uncertainty among the MLIP ensemble with each generation, as seen in Figure S20a.2–f.2. We report MAE for the reaction PES, along with errors in forward and reverse barriers in Table 4. Overall, the agreement between the MLIP predictions with the DFT reaction pathways is quite good. Reactions 4 and 6 have high MAE in energy prediction and somewhat higher errors in the PES and the forward barriers. Reaction 4 is a C–C coupling reaction (CH_3(ads)_ + CH_2(ads)_ + H_(ads)_ → C_2_H_5(ads)_ + H_(ads)_) and reaction 6 is an ethane formation reaction (C_2_H_5(ads)_ + H_(ads)_ → C_2_H_6_). The accuracy of our predictions would increase if we had performed more extensive training.

**Figure 7:**
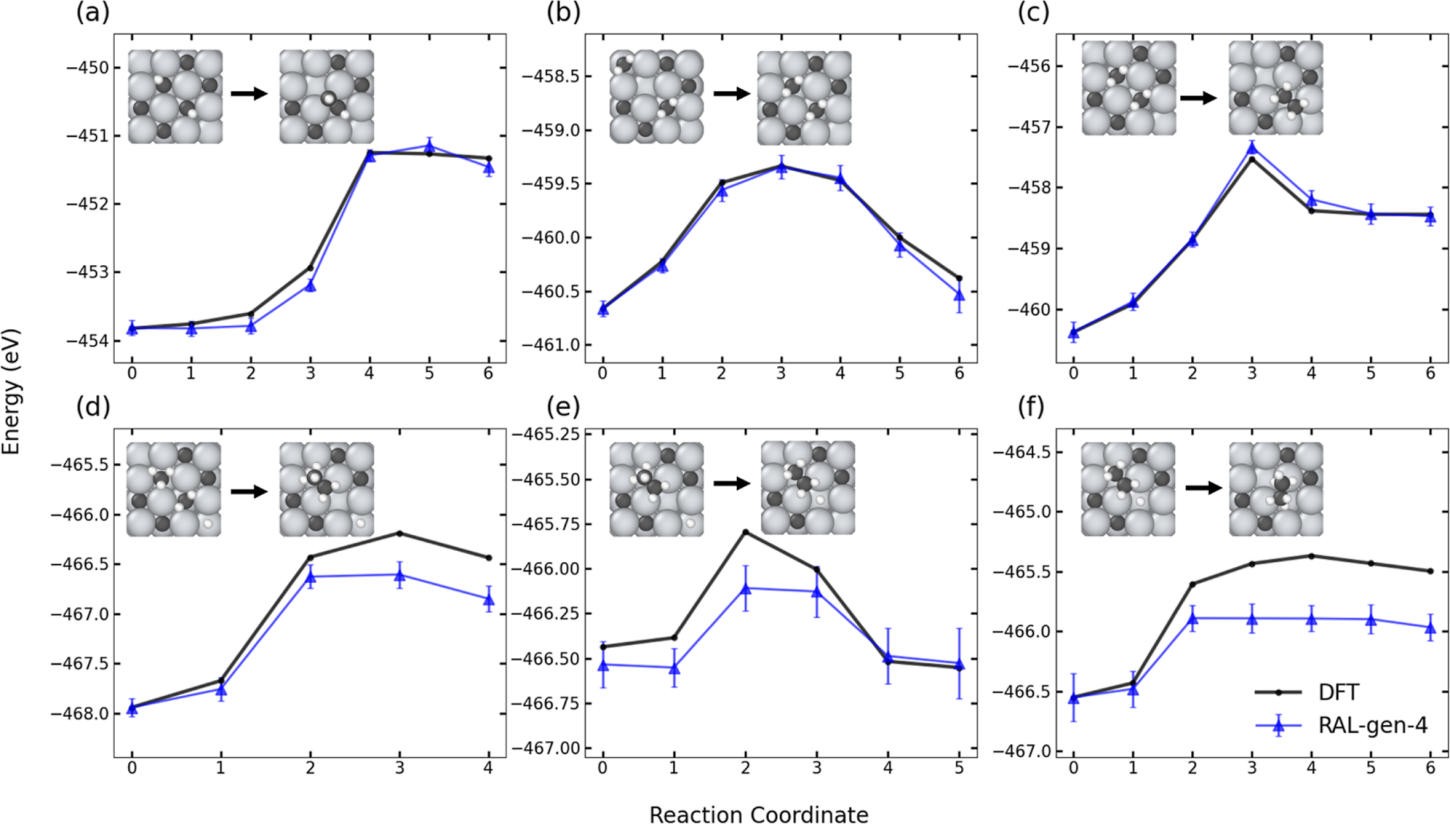
TiC heterogeneous catalysis benchmarking reactions with RAL gen-4 (blue diamonds) compared with DFT (black circles). The error bars represent the largest and smallest predicted energies across four MLIPs.

**Table 4:** Mean Absolute Error (MAE) for the reaction PES and Forward and Reverse Barrier Predictions by Gen-4 MLIP of the TiC System^a^

model	RAL gen-4 (eV)
reaction 1	0.11 (0.09, 0.23)
reaction 2	0.05 (0.01, 0.14)
reaction 3	0.07 (0.20, 0.22)
reaction 4	0.22 (0.42, 0.00)
reaction 5	0.13 (0.22, 0.34)
reaction 6	0.32 (0.52, 0.05)

a All results are reported in eV.

### 3.5 Methane Reactions on Ti_*x*_C_*y*_ Surfaces

We have used the final MLIP for the Ti_*x*_C_*y*_ systems to explore adsorption and reaction of CH_4_ on various Ti_*x*_C_*y*_ surfaces. Distinct clean surfaces are shown in Figure 8. Note that there are two different Ti_8_C_5_(001) terminations, one Ti-terminated and one C-terminated, shown in Figure 8c,d, respectively. The final snapshots from 500 ps simulations for each of these three systems are also shown in Figure 8. Note that the snapshot for TiC mainly shows physisorbed CH_4_. The Ti_2_C surface exhibits chemisorbed carbon and hydrogen, which we denote as C* and H*. The Ti_8_C_5_ surfaces show mainly CH* and H*.

**Figure 8:**
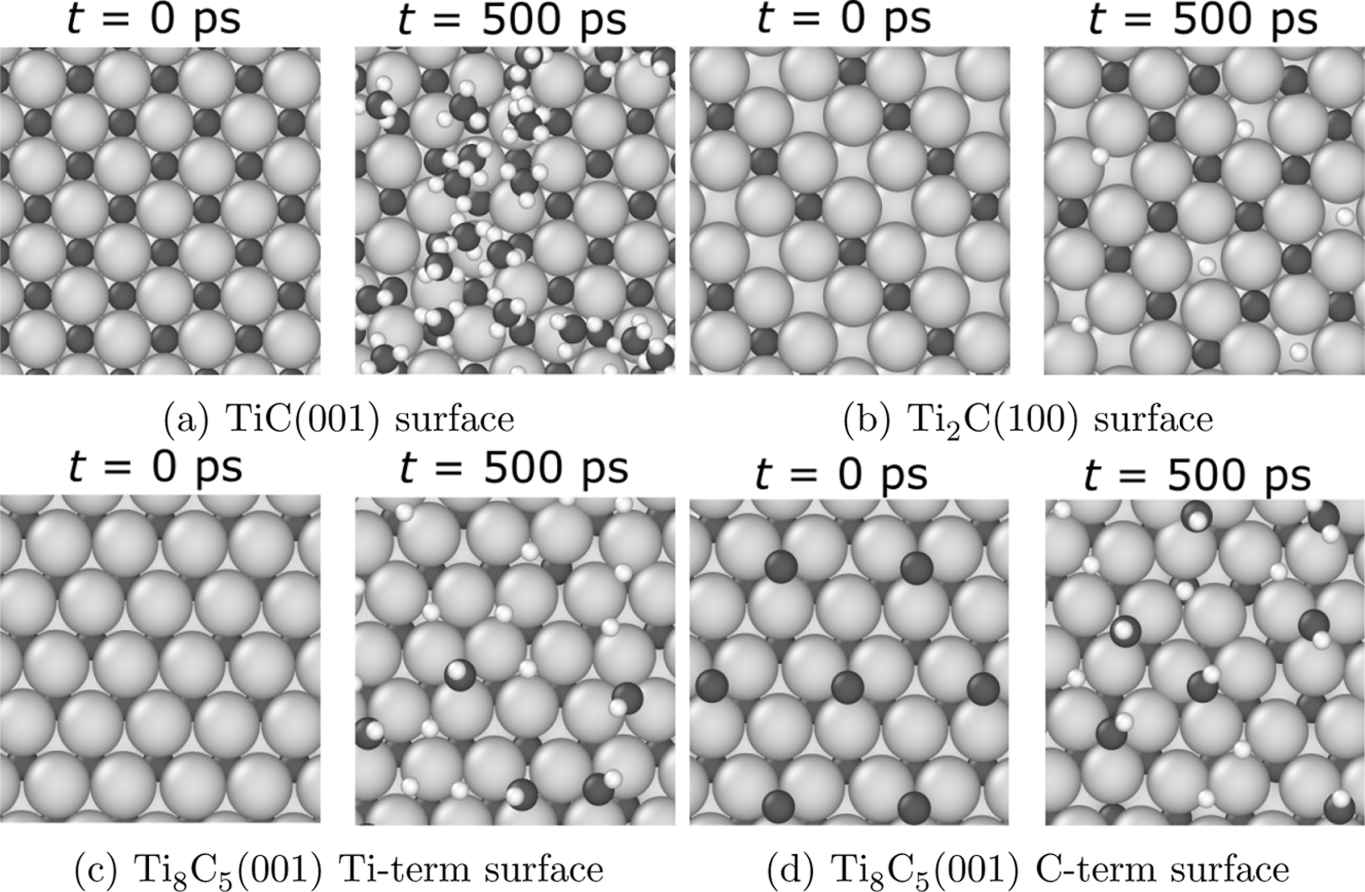
Snapshots of Ti_*x*_C_*y*_ surfaces before (*t* = 0 ps) and after (*t* = 500 ps) *T* = 1200 K *NVT*-MD simulations initialized with 20 CH_4_ molecules. Ti atoms shown in light gray, C in dark gray, H in white.

The time dependence of the number of unreacted CH_4_ molecules on these three surfaces is shown in Figure 9a. We see that the TiC(001) surface, which is a fully coordinated stoichiometry of titanium carbide, is almost unreactive. Only about 10% of the CH_4_ molecules decompose over the course of the simulation. The slow rate of reaction can be attributed to the absence of undercoordinated Ti atoms, which act as active sites. The Ti_8_C_5_(001) surface has a C/Ti ratio of 0.625 and therefore has a significant number of undercoordinated Ti sites. Over 60% of the CH_4_ molecules decomposed on the Ti_8_C_5_ surface by the end of the simulation. The Ti_2_C­(100) surface exhibits the highest reactivity, decomposing over 90% of the CH_4_ molecules. For Ti_2_C, Ti has three neighboring carbon atoms, inducing multiple C vacancies on the surface. These vacancies are responsible for the reactivity of the Ti_2_C surface, as seen at the end of the simulation at *t* = 500 ps. The decomposition rate decreases dramatically after about 100 ps for both Ti_8_C_5_ and Ti_2_C. This decrease in rate is due to two factors: (1) the decrease in the number of reactant CH_4_ molecules, and (2) the number of active sites available on the surfaces decreases due to the blocking of Ti sites by chemisorbed CH_x_* and H* atoms.

**Figure 9:**
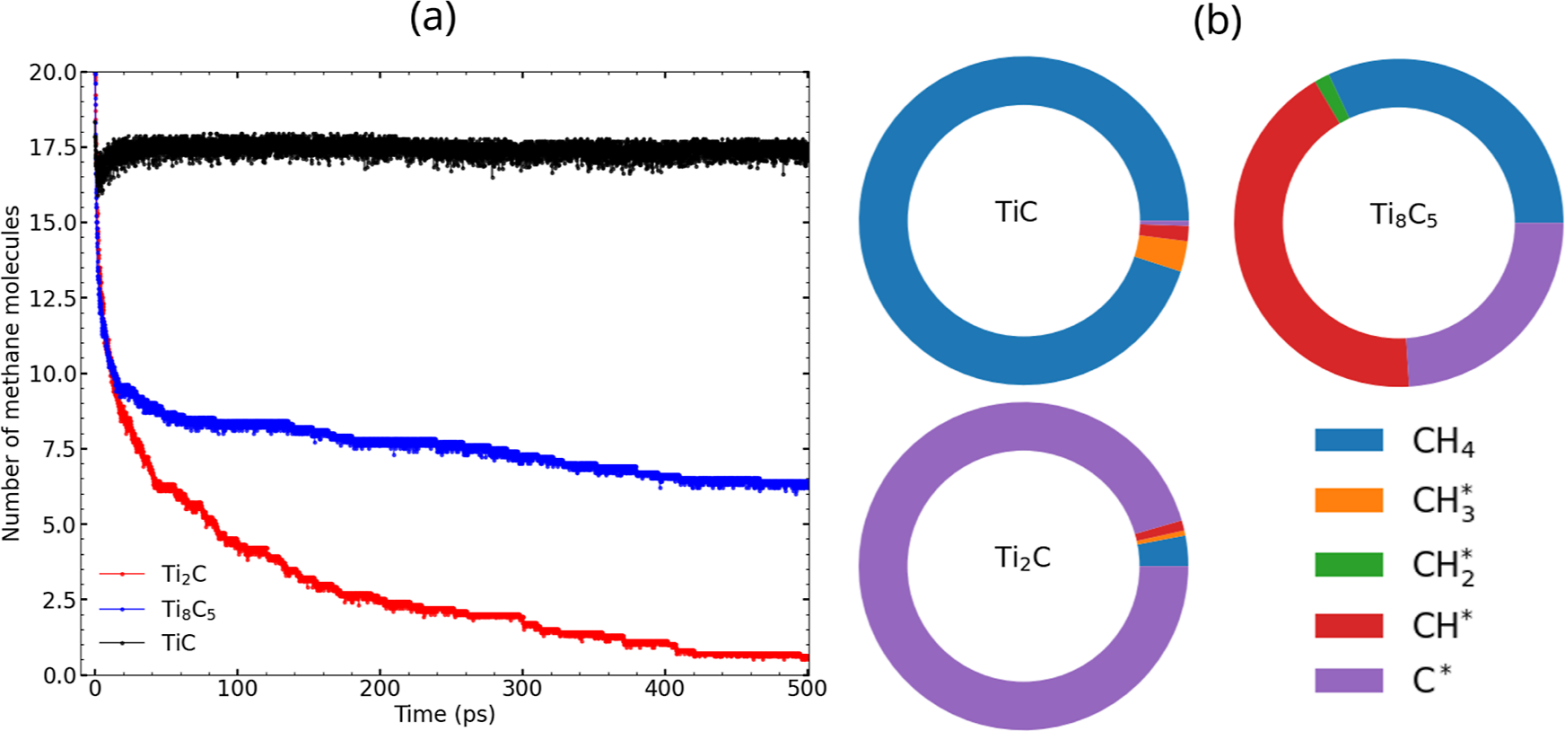
(a) Evolution of CH_4_ decomposition with time for *NVT* simulations at 1200 K starting with 20 CH_4_ molecules on three different Ti_*x*_C_*y*_ surfaces. (b) Fractions of different carbon species adsorbed on the three surfaces at the end of a 500 ps simulation. All results are averages over 10 independent runs.

To investigate the impact of surface structure on the kinetics of the CH_4_ decomposition reaction, we measured initial rates (in molecules/ps) of CH_4_ decomposition on the Ti_2_C­(100) and Ti_8_C_5_(001) surfaces. The initial rates are plotted in Figure S21. Both Ti_2_C and Ti_8_C_5_ displayed initial “dead times”, during which the number of CH_4_ molecules remained constant for a few hundred fs. This dead time is due to CH_4_ molecules diffusing to the surface from the bulk (vacuum space) and physisorbing on the Ti_*x*_C_*y*_ surfaces. The magnitude of the initial decomposition rate is higher on Ti_8_C_5_ than on Ti_2_C. This could be explained by the types of active sites on Ti_2_C and Ti_8_C_5_. There are three types of active sites on Ti_2_C: (i) Ti atop, (ii) bridge sites, and (iii) hollow sites due to C vacancies. Note that the atop and bridge sites do not appear to be active for dissociation because the decomposition products are observed essentially only on the hollow sites. On the other hand, Ti_8_C_5_ has more types of active surface sites than Ti_2_C, due to having two different terminations. In addition to atop sites, which do not appear to be active as judged by the location of chemisorbed species, there are two types of 3-fold sites, bridge sites, and surface C sites that are populated with decomposition products.

Arrhenius plots for Ti_2_C and Ti_8_C_5_ are shown in Figure 10. For all temperatures, the initial rates for Ti_8_C_5_ are higher than Ti_2_C. The apparent activation energy value, Δ*E*_app_ for Ti_2_C­(100) is similar to the value of 0.23 eV for CH_4_ to CH_3_* activation energy computed from DFT, as reported in Pal et al.^63^ We believe this is a fortuitous agreement, since the value we calculated is an effective rate for all dissociation events. The calculated value for Ti_8_C_5_ is smaller than Ti_2_C by 0.11 eV, confirming that the initial dissociation kinetics is faster on Ti_8_C_5_ than on Ti_2_C. We are not aware of any reports for CH_4_ reactions on Ti_8_C_5_ surfaces; hence, the value for apparent activation energy could not be compared to any values from the literature.

**Figure 10:**
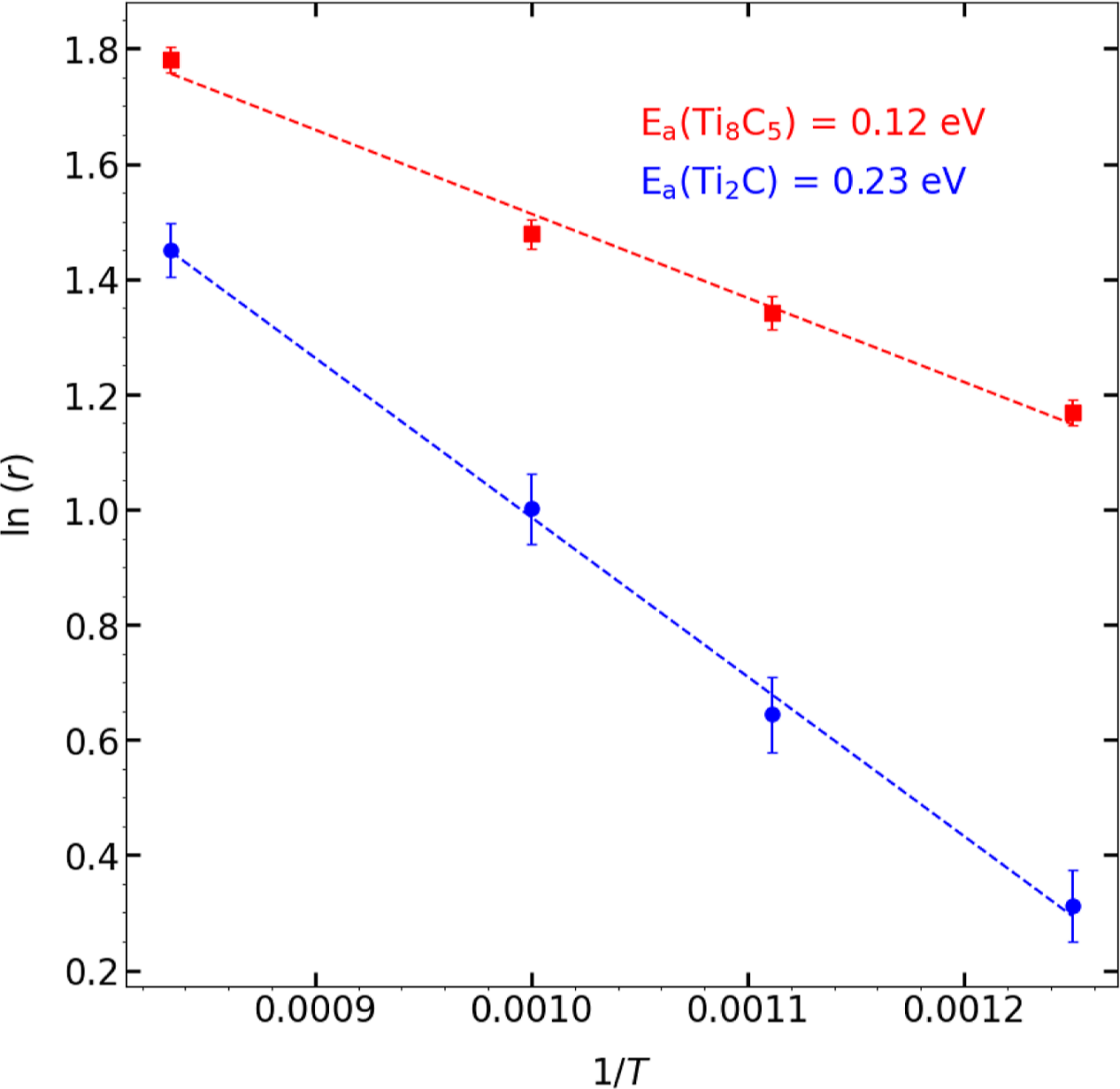
ln­(*r*) vs 1/*T* plot for Ti_2_C and Ti_8_C_5_. The initial rate *r* is in molecules/ps and *T* in K. The ln­(*r*) is larger for Ti_8_C_5_, and the overall apparent activation energy is lower for Ti_8_C_5_ than Ti_2_C. Dashed lines are linear fits to the data. The error bars represent two standard deviations of the mean of the rates.

We note that the CH_4_ dissociation products are different on Ti_2_C and Ti_8_C_5_. The intermediate product distributions at 500 ps are shown in Figure 9b. The most abundant surface species for Ti_2_C is C*, and for Ti_8_C_5_ it is CH* and C*. The CH_2_* and CH_3_* species coverages are essentially negligible on Ti_2_C, whereas for Ti_8_C_5_ CH_2_* is present in significant amounts. The C* fraction on Ti_8_C_5_ is lower than the CH* fraction, suggesting a low extent of complete CH_4_ decomposition.

To explore reaction rates and surface coverages on Ti_2_C at longer times and higher CH_4_ concentrations, we performed two continuation simulations by sequentially dosing the system with 20 additional CH_4_ molecules in the bulk at the beginning of each run. Specifically, we started the second simulation using the ending configuration from the Ti_2_C simulation shown as the red curve in Figure 9a and added 20 additional CH_4_ molecules to the bulk. *NVT*-MD was carried out at 1200 K for another 500 ps. At the end of that run, we again dosed with 20 CH_4_ molecules and ran for another 500 ps. We calculated the C/Ti, H/Ti, and [C + H]/Ti ratios by counting the number of C, and H atoms on the surface as a function of time for all three simulations. These ratios are shown in Figure 11. The initial C/Ti ratio before initial dosing was 0.5, as required by the Ti_2_C surface. It increases to 0.58 at the end of the initial dosing, 0.6 at the end of the second dosing, and 0.64 at the end of the third dosing. In all dosing stages, adsorbed species remain on the surface with no desorption. The decomposition of CH_4_ generates hydrogen atoms, which occupy other surface sites. The H/Ti ratio at the end of the third dosing is 0.35. Hence, the [C + H]/Ti ratio approaches 1 at the end of the third simulation.

**Figure 11:**
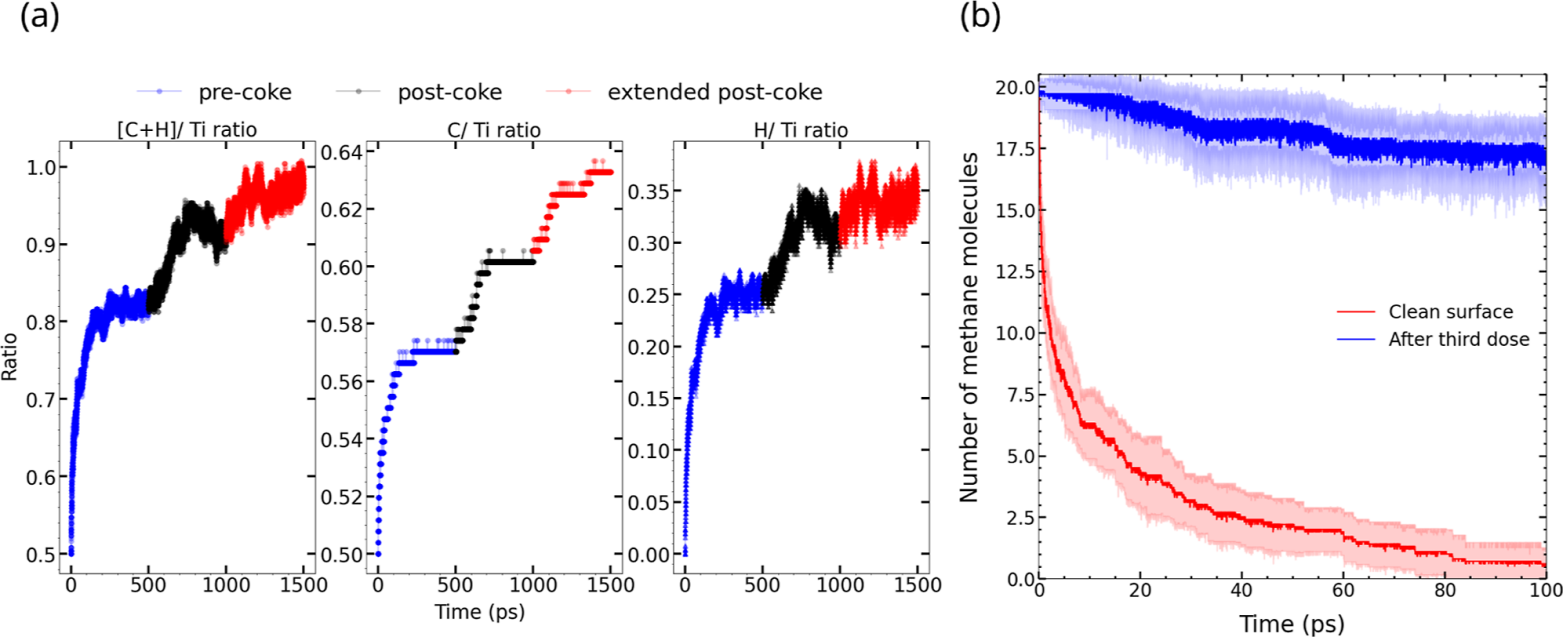
(a) Ratios of surface C to surface Ti atoms (C/Ti), surface H to surface Ti atoms (H/Ti), and combined surface C and H atoms over surface Ti ([C + H]/Ti) as a function of time for three consecutive simulations, each starting with 20 CH_4_ molecules. The minimum C/Ti ratio is 0.5, which is the stoichiometric ratio for Ti_2_C. The C/Ti ratio increases with loading and reaches a value of about 0.65. The combined [C + H]/Ti ratio approaches 1, indicating complete occupation of surface sites. The simulation temperature was 1200 K. (b) Number of unreacted CH_4_ molecules vs time from *NVT*-MD simulations at 1200 K for the clean Ti_2_C surface and for the Ti_2_C surface at the end of 3 doses of 20 CH_4_ molecules.

The evolution of carbon dissociation products with simulation time is shown in Figure S22a,b. In the first 100 ps, CH_4_ molecules rapidly decompose to form CH_3_*, CH_2_*, CH*, and C*. All CH_x_* species are rapidly decomposed to C*. The short lifetime of these intermediates indicates a low possibility of coupling reactions on Ti_2_C. The major intermediate, C*, did not desorb during later dosing stages, as observed from C/Ti ratios. The strong adsorption can be explained by the binding energy. The rate of CH_4_ decomposition reduced after the second dose in the Ti_2_C system, with only 25% of bulk CH_4_ converting to surface species. The major surface species during the second dose was C*. This observation is consistent with Kuriakose et al., as they reported the CH_4_ activation barrier is smaller than the C_2_H_2_ formation barrier for the Ti_2_C­(100) surface.^61^ Indeed, we did not observe any coupling products in the given simulation time on Ti_2_C­(100).

We characterized surface deactivation by comparing initial rates on the Ti_2_C surface for the initial dose with the initial rate after three doses, where the surface is covered with C* and H*. The initial rate for the clean surface was significantly higher (2.34 molecules/ps) than for the surface after the third dose (0.03 molecules/ps), as shown in Figure 11b. The rate of CH_4_ decomposition on the surface covered with C* and H* can be compared with the TiC surface, which was almost inactive toward CH_4_ decomposition. The initial rate calculation showed that the Ti_2_C surface is very rapidly poisoned by CH_4_ decomposition products and must be regenerated by desorption of C* and H*. We observed diffusion of surface hydrogen and generation of H_2_ in simulations with high-surface coverage, indicating that some surface sites can be regenerated at high temperatures.

Figure S22c shows the evolution of carbon dissociation products with simulation time in Ti_8_C_5_(001). As mentioned previously in Figure 9b, Ti_8_C_5_ has CH* and C* in significant proportion. However, along with decomposition intermediates, we see C–C coupling intermediates formed on the C-terminated surface, but not the Ti-terminated surface, as shown in Figure S23. We observed C_2_*, C_2_H*, C_3_H*, C_2_H_2_* as some of the coupling products. Even though coupling intermediates are present in a smaller fraction when compared with decomposition intermediates, the presence of these intermediates show the capability of Ti_8_C_5_ to form coupling products.

## 4 Conclusion

We have demonstrated that the RAL framework can efficiently train MLIPs to account for chemical reactions with near-DFT accuracy. RAL differs from other training methods in that it uses methods to automatically generate reaction products based only on specifying reactants and then automatically produces commands to use partially trained MLIPs to explore reaction pathways through reaction-explicit active learning. Reaction pathways are explicitly sampled through transition state finding methods, such as SE-GSM, using the MLIP that is being trained to perform reaction exploration AL. We have shown that identifying just a few possible reaction products can lead to hundreds of individual reaction pathways for use in RAL. Reaction intermediates and products are automatically discovered, and MLIPs are trained with MD-AL to learn these new chemical species during the RAL process. Thus, the user does not need to provide predefined pathways or even a list of potential products.

We demonstrated the versatility of RAL by applying the method to gas-phase ammonia synthesis, methanimine hydrolysis, and to methane activation on Ti_*x*_C_*y*_ surfaces, showing that RAL can be used for homogeneous and heterogeneous systems. We have compared the ability of RAL-trained MLIPs to predict reaction pathways not included in the training set. We find that the overall pathway is predicted with good accuracy. However, the reaction barriers are typically not predicted with high accuracy. This is likely due to incomplete sampling of TS and near-TS structures. Because of their highly transient nature, these structures are not sampled in MD-AL and are only included in the form of single structures as relabeled data. Improving TS sampling within the RAL framework may provide a route to quantitative reaction barrier estimation.

It is important to put the accuracy of the RAL predictions for PES and barrier heights in context. The sets of benchmark reactions with which we compared were not included in the training sets and therefore are true predictions. Of the MLIPs in the literature trained to account for chemical reactions, several were not evaluated by comparing with computed barriers with quantum chemical calculations.^17,20–22^ Other MLIPs were trained to account for specific reactions, so comparison with quantum chemical calculations in these cases cannot be considered true predictions.^12–16,18^ In these cases, MLIPs can very accurately reproduce the reference PES for the reactions on which they were trained. For example, Schaaf et al.^19^ trained an MLIP to reproduce energy barriers for CO_2_ hydrogenation to CH_3_OH on an indium oxide surface within 0.05 eV of DFT values. In a similar approach, Guo et al.^23^ studied C–H bond activation within zeolites having [CuOCu]^2+^ sites. They included 5446 configurations in their training and then predicted barriers for similar [CuOCu]^2+^ sites on other zeolites. Their MLIPs gave a MAE of 0.07 eV compared with DFT calculations on 3356 sites. This is remarkable accuracy, but cannot be considered a true prediction because the [CuOCu]^2+^ sites are similar in the different zeolites. On the other hand, Lee et al.^24^ predicted energy barriers for 11,961 TS structures from MLIPs trained on a data set containing 85 reactants with a total of 417,455 data points. They achieved an MAE of about 0.66 eV in the TS barriers for the predicted reactions. Our accuracy for predicted TS barriers is similar to that of Lee et al., with an MAE of 0.5 eV including all barriers. We conclude from this assessment of RAL in comparison with the literature methods that if one requires very high accuracy in reaction barriers that the MLIP must be trained with the specific reaction pathways or intermediates. If, on the other hand, one does not have a priori knowledge of the reaction pathways or even the reaction products, RAL is a method that can discover intermediates and products with reasonable accuracy.

RAL is a practical recipe for developing reactive MLIPs. It provides a systematic workflow to (1) balance exploration of chemical and configurational spaces through iterative uncertainty sampling; (2) automate reaction discovery via product prediction and TS search tools (e.g., SE-GSM, NEB); (3) validate MLIPs using quantitative metrics (force deviations, convex hull volumes, cumulative NN distances). The framework’s architecture-agnostic design makes it adaptable to any MLIP using any level of quantum chemistry that is feasible for the problem. We also note that enhanced sampling techniques, such as metadynamics, could also be incorporated, if the user has specific known reactions to be included in the training.

Finally, we make some recommendations for practical implementation of the RAL formalism. 1. We do not claim to know the best balance of reaction exploration (sampling chemical space) and MD-AL (sampling configurational space). We have used NSC-AL, SC-AL, and a hybrid of the two. What is clear is that some MD-AL is needed to effectively learn chemical intermediates and products discovered during RAL cycles. In this work, which is a proof-of-concept exploration, we learned as we tried different methods. On balance, we recommend SC-AL as the most robust choice because we found that for methanimine the NSC-AL method did not converge within a reasonable number of generations. Hence, while SC-AL imposes a higher computational cost through additional MLIP training, it has a higher chance of converging for difficult systems. 2. It could happen that the bootstrap MLIP for a specific system will not be trained well enough to allow for efficient reaction path sampling in gen-1. This was the case for the Ti_*x*_C_*y*_ system in our study, but that did not turn out to be critical, since even the few reactions that were filtered were sufficient to allow for efficient sampling in gen-2.3. We have used only a single criterion to assess convergence of the MLIP, the maximum force deviation. Higher accuracy could be achieved by reducing the convergence criterion from 0.5 eV/Å to a lower value. In hindsight, we think it would be useful to use the convex hull volumes, which should be large throughout all generations, and the cumulative NN distances, which should decay toward zero for more complete explorations of the chemical and configurational spaces, as additional criteria to judge how well the MLIPs are converging. Other convergence criteria could also be employed.

## Supplementary Material



## Data Availability

The codes for RAL used in this study are publicly available at: https://github.com/siddarthachar/reactive_active_learning.

## Notes

Details of the density functional theory method, the ML potential, the transition state finding method, the evaluation metrics, and the Nanoreactor active learning training; kernel density estimates of all systems and training types; UMAP plots for all systems and training types; predictions of intermediate energies for different generations of all systems; convex hull and cumulative nearest neighbors (NN) distances for all generations of all systems and training types; comparison of compute resources (SUs) used for RAL and NR-AL; decomposition of methane molecules and evolution of decomposition products on Ti_*x*_C_*y*_ surfaces (PDF)

## #

Pritzker School of Molecular Engineering, University of Chicago, Chicago, Illinois 60637, United States

## References

[1] van
Duin A. C. T., Dasgupta S., Lorant F., Goddard W. A. (2001). ReaxFF:
A Reactive Force Field for Hydrocarbons. J.
Phys. Chem. A.

[2] Senftle T. P., Hong S., Islam M. M., Kylasa S. B., Zheng Y., Shin Y. K., Junkermeier C., Engel-Herbert R., Janik M. J., Aktulga H. M. (2016). The ReaxFF reactive
force-field: development, applications and future directions. npj Comput. Mater..

[3] Merchant A., Batzner S., Schoenholz S. S., Aykol M., Cheon G., Cubuk E. D. (2023). Scaling Deep Learning for Materials Discovery. Nature.

[4] Gelžinytė E., Öeren M., Segall M. D., Csányi G. (2024). Transferable
Machine Learning Interatomic Potential for Bond Dissociation Energy
Prediction of Drug-like Molecules. J. Chem.
Theory Comput..

[5] Gkeka P., Stoltz G., Barati Farimani A., Belkacemi Z., Ceriotti M., Chodera J. D., Dinner A. R., Ferguson A. L., Maillet J.-B., Minoux H. (2020). Machine Learning Force
Fields and Coarse-Grained Variables in Molecular Dynamics: Application
to Materials and Biological Systems. J. Chem.
Theory Comput..

[6] Omranpour A., Elsner J., Lausch K. N., Behler J. (2025). Machine Learning Potentials
for Heterogeneous Catalysis. ACS Catal..

[7] Olajide G., Baral K., Ezendu S., Soyemi A., Szilvási T. (2025). Application
of machine learning interatomic potentials in heterogeneous catalysis. J. Catal..

[8] Zhang Y., Wang H., Chen W., Zeng J., Zhang L., Wang H., Weinan E. (2020). DP-GEN: A concurrent learning platform
for the generation of reliable deep learning based potential energy
models. Comput. Phys. Commun..

[9] Yang Y., Zhang S., Ranasinghe K. D., Isayev O., Roitberg A. E. (2024). Machine
Learning of Reactive Potentials. Annu. Rev.
Phys. Chem..

[10] Young T. A., Johnston-Wood T., Deringer V. L., Duarte F. (2021). A transferable active-learning
strategy for reactive molecular force fields. Chem. Sci..

[11] Young T. A., Johnston-Wood T., Zhang H., Duarte F. (2022). Reaction dynamics of
Diels–Alder reactions from machine learned potentials. Phys. Chem. Chem. Phys..

[12] Devergne T., Magrino T., Pietrucci F., Saitta A. M. (2022). Combining Machine
Learning Approaches and Accurate Ab Initio Enhanced Sampling Methods
for Prebiotic Chemical Reactions in Solution. J. Chem. Theory Comput..

[13] David R., Tuñón I., Laage D. (2024). Competing Reaction Mechanisms of
Peptide Bond Formation in Water Revealed by Deep Potential Molecular
Dynamics and Path Sampling. J. Am. Chem. Soc..

[14] David R., de la Puente M., Gomez A., Anton O., Stirnemann G., Laage D. (2025). ArcaNN: automated enhanced sampling generation of training sets for
chemically reactive machine learning interatomic potentials. Digital Discovery.

[15] Guan X., Heindel J. P., Ko T., Yang C., Head-Gordon T. (2023). Using machine
learning to go beyond potential energy surface benchmarking for chemical
reactivity. Nat. Comput. Sci..

[16] Zeng Z., Wodaczek F., Liu K., Stein F., Hutter J., Chen J., Cheng B. (2023). Mechanistic insight on water dissociation
on pristine low-index TiO_2_ surfaces from machine learning
molecular dynamics simulations. Nat. Commun..

[17] Gomez A., de la Puente M., David R., Laage D. (2024). Neural network potentials
for exploring condensed phase chemical reactivity. C. R. Chim..

[18] Zhang P., Gardini A. T., Xu X., Parrinello M. (2024). Intramolecular
and Water Mediated Tautomerism of Solvated Glycine. J. Chem. Inf. Model..

[19] Schaaf L. L., Fako E., De S., Schäfer A., Csányi G. (2023). Accurate energy barriers for catalytic reaction pathways:
an automatic training protocol for machine learning force fields. npj Comput. Mater..

[20] Zhang S., Makoś M. Z., Jadrich R. B., Kraka E., Barros K., Nebgen B. T., Tretiak S., Isayev O., Lubbers N., Messerly R. A. (2024). Exploring the frontiers of condensed-phase
chemistry with a general reactive machine learning potential. Nat. Chem..

[21] Zeng J., Cao L., Xu M., Zhu T., Zhang J. Z. H. (2020). Complex reaction
processes in combustion unraveled by neural network-based molecular
dynamics simulation. Nat. Commun..

[22] Zeng J., Zhang L., Wang H., Zhu T. (2021). Exploring the Chemical
Space of Linear Alkane Pyrolysis via Deep Potential GENerator. Energy Fuels.

[23] Guo J., Sours T., Holton S., Sun C., Kulkarni A. R. (2024). Screening
Cu-Zeolites for Methane Activation Using Curriculum-Based Training. ACS Catal..

[24] Lee M., Ucak U. V., Jeong J., Ashyrmamatov I., Lee J., Sim E. (2025). Automated and Efficient Sampling of Chemical Reaction
Space. Adv. Sci..

[25] Laio A., Parrinello M. (2002). Escaping free-energy minima. Proc. Natl. Acad. Sci. U.S.A..

[26] Kästner J. (2011). Umbrella sampling. Wiley Interdiscip. Rev. Comput. Mol. Sci..

[27] Dellago, C. ; Bolhuis, P. G. ; Geissler, P. L. Transition Path Sampling. In Advances in Chemical Physics; John Wiley & Sons, Ltd, 2002, pp 1–78. Chapter 1.

[28] Kulichenko M., Barros K., Lubbers N., Li Y. W., Messerly R., Tretiak S., Smith J. S., Nebgen B. (2023). Uncertainty-driven
dynamics for active learning of interatomic potentials. Nat. Comput. Sci..

[29] Invernizzi M., Parrinello M. (2020). Rethinking Metadynamics: From Bias Potentials to Probability
Distributions. J. Phys. Chem. Lett..

[30] Henkelman G., Jónsson H. (2000). Improved Tangent Estimate in the Nudged Elastic Band
Method for Finding Minimum Energy Paths and Saddle Points. J. Chem. Phys..

[31] Henkelman G., Uberuaga B. P., Jónsson H. (2000). A Climbing Image Nudged Elastic Band
Method for Finding Saddle Points and Minimum Energy Paths. J. Chem. Phys..

[32] Sheppard D., Terrell R., Henkelman G. (2008). Optimization Methods for Finding
Minimum Energy Paths. J. Chem. Phys..

[33] Wang H., Zhang L., Han J., E W. (2018). DeePMD-Kit: A Deep Learning Package
for Many-Body Potential Energy Representation and Molecular Dynamics. Comput. Phys. Commun..

[34] Yoo P., Sakano M., Desai S., Islam M. M., Liao P., Strachan A. (2021). Neural network reactive force field for C, H, N, and
O systems. npj Comput. Mater..

[35] Schreiner M., Bhowmik A., Vegge T., Busk J., Winther O. (2022). Transition1x-a
dataset for building generalizable reactive machine learning potentials. Sci. Data.

[36] Chai J.-D., Head-Gordon M. (2008). Long-range corrected hybrid density functionals with
damped atom–atom dispersion corrections. Phys. Chem. Chem. Phys..

[37] Schütt, K. ; Unke, O. ; Gastegger, M. Equivariant message passing for the prediction of tensorial properties and molecular spectra. In Proceedings of the 38th International Conference on Machine Learning, 2021; pp 9377–9388.

[38] Grambow C. A., Pattanaik L., Green W. H. (2020). Reactants, products, and transition
states of elementary chemical reactions based on quantum chemistry. Sci. Data.

[39] Zimmerman P. M. (2015). Single-ended
transition state finding with the growing string method. J. Comput. Chem..

[40] Bannwarth C., Ehlert S., Grimme S. (2019). GFN2-xTB—An Accurate and Broadly
Parametrized Self-Consistent Tight-Binding Quantum Chemical Method
with Multipole Electrostatics and Density-Dependent Dispersion Contributions. J. Chem. Theory Comput..

[41] Seung, H. S. ; Opper, M. ; Sompolinsky, H. Query by committee. In Proceedings of the Fifth Annual Workshop on Computational Learning Theory; ACM: New York, NY, USA, 1992; pp 287–294.

[42] Tu Z., Coley C. W. (2022). Permutation Invariant Graph-to-Sequence Model for Template-Free
Retrosynthesis and Reaction Prediction. J. Chem.
Inf. Model..

[43] Boes J. R., Mamun O., Winther K., Bligaard T. (2019). Graph Theory Approach
to High-Throughput Surface Adsorption Structure Generation. J. Phys. Chem. A.

[44] Evans D. J., Holian B. L. (1985). The Nose–Hoover thermostat. J. Chem. Phys..

[45] Kresse G., Hafner J. (1993). Ab Initio Molecular Dynamics for Liquid Metals. Phys. Rev. B.

[46] Kresse G., Hafner J. (1994). Ab Initio Molecular-Dynamics Simulation of the Liquid-Metal–Amorphous-Semiconductor
Transition in Germanium. Phys. Rev. B.

[47] Kresse G., Furthmüller J. (1996). Efficiency of ab-initio total energy calculations for
metals and semiconductors using a plane-wave basis set. Comput. Mater. Sci..

[48] Kresse G., Furthmüller J. (1996). Efficient Iterative Schemes for Ab Initio Total-Energy
Calculations Using a Plane-Wave Basis Set. Phys.
Rev. B.

[49] Weininger D. (1988). SMILES, a
chemical language and information system. 1. introduction to methodology
and encoding rules. J. Chem. Inf. Comput. Sci..

[50] Zhao Q., Savoie B. M. (2021). Simultaneously improving reaction coverage and computational
cost in automated reaction prediction tasks. Nat. Comput. Sci..

[51] Larsen A. H., Mortensen J. J., Blomqvist J., Castelli I. E., Christensen R., Dułak M., Friis J., Groves M. N., Hammer B., Hargus C. (2017). Atomic Simulation Environment—a Python Library
for Working with Atoms. J. Phys.: Condens. Matter.

[52] Bitzek E., Koskinen P., Gähler F., Moseler M., Gumbsch P. (2006). Structural
Relaxation Made Simple. Phys. Rev. Lett..

[53] Martínez L., Andrade R., Birgin E. G., Martínez J. M. (2009). PACKMOL:
A package for building initial configurations for molecular dynamics
simulations. J. Comput. Chem..

[54] Healy J., McInnes L. (2024). Uniform manifold approximation and projection. Nat. Rev. Methods Primers.

[55] Hwang D.-Y., Mebel A. M. (2003). Reaction Mechanism of N2/H2 Conversion to NH3: A Theoretical
Study. J. Phys. Chem. A.

[56] Deng L., Ziegler T. (1994). The determination of intrinsic reaction coordinates
by density functional theory. Int. J. Quantum
Chem..

[57] Ali M. A. (2020). Computational
studies on the gas phase reaction of methylenimine (CH_2_NH) with water molecules. Sci. Rep..

[58] Kunkel C., Viñes F., Illas F. (2018). Biogas Upgrading by Transition Metal
Carbides. ACS Appl. Energy Mater..

[59] Wan W., Tackett B. M., Chen J. G. (2017). Reactions of water and C1 molecules
on carbide and metal-modified carbide surfaces. Chem. Soc. Rev..

[60] de
Jong M., Chen W., Angsten T., Jain A., Notestine R., Gamst A., Sluiter M., Krishna Ande C., van der Zwaag S., Plata J. J. (2015). Charting the complete
elastic properties of inorganic crystalline compounds. Sci. Data.

[61] Kuriakose N., Mondal U., Ghosh P. (2021). CH_4_ Activation and C–C
Coupling on the Ti_2_C (100) Surface in the Presence of Intrinsic
C-Vacancies: Is Excess Good?. J. Mater. Chem.
A.

[62] Giannozzi P., Baroni S., Bonini N., Calandra M., Car R., Cavazzoni C., Ceresoli D., Chiarotti G. L., Cococcioni M., Dabo I. (2009). QUANTUM ESPRESSO: a
modular and open-source software project for quantum simulations of
materials. J. Phys.: Condens. Matter.

[63] Pal R., Ghosh P. (2023). C-Vacancy Mediated Methane Activation and C–C Coupling on
TiC(001) Surfaces: A First-Principles Investigation. J. Phys. Chem. C.

