# Supplementary material for: Reactive Active Learning: An Efficient Approach for Training Machine Learning Interatomic Potentials for Reacting Systems
Source: J Chem Theory Comput. 2025 Sep 3;21(18):8889–906. doi: 10.1021/acs.jctc.5c00920 (PMC12487985; doi:10.1021/acs.jctc.5c00920)
Supplement: Supplementary file 1 [file ct5c00920_si_001.pdf]

Supporting Information: Reactive Active Learning: An Efficient Approach for Training Machine Learning Interatomic Potentials for Reacting Systems

Siddarth K. Achar,[†,‖] Priyanka B. Shukla,[‡] Chinmay V. Mhatre,[‡] Leonardo Bernasconi,[¶] Caitlyn Y. Vinger,[§] and J. Karl Johnson[*,‡]

†*Computational Modeling & Simulation Program, University of Pittsburgh, Pittsburgh, PA, 15260, United States*

‡*Department of Chemical & Petroleum Engineering, University of Pittsburgh, Pittsburgh, PA, 15261, United States*

¶*Center for Research Computing and Data, University of Pittsburgh, Pittsburgh, PA, 15260, United States*

§*Department of Electrical & Computer Engineering, University of Pittsburgh, Pittsburgh, PA, 15261, United States*

‖*Current address: Pritzker School of Molecular Engineering, University of Chicago, Chicago, Illinois 60637, United States*

E-mail: karlj@pitt.edu

S1 Density functional theory

We used density functional theory (DFT) using the Vienna *ab initio* simulation package (VASP)[S1–S4] to represent our "ground truth" data for three reactions: (i) uncatalyzed ammonia synthesis, (ii) methanimine hydrolysis, and (iii) methane activation and C–C coupling on Ti_xC_y surfaces. The first two were modeled in the homnogeneous phase, while the last was modeled as a heterogeneous catalysis system. For all three systems, electron–ion interactions were described using the projector-augmented wave (PAW) method,[S5] and exchange-correlation effects were treated with the Perdew–Burke–Ernzerhof (PBE) functional.[S6,S7] Van der Waals interactions were included using the DFT-D3 method with the Becke-Johnson damping function.[S8] Only the Γ point was used for Brillouin zone sampling, as these systems involve isolated molecules for the homogeneous reactions. We account for spin-polarization for these reactions by initializing the magnetic moment of each atom to zero. Configurations with non-integer final magnetic moments (within a tolerance of 10^{-3} μ_B) were discarded. For methane activation and C–C coupling on Ti_xC_y surfaces, periodic slab models were constructed, and DFT calculations were carried out using a Monkhorst-Pack k-point mesh with a 0.5 Å spacing in the Brillouin zone.

S2 Machine Learning (ML) Potential

The DeePMD formalism was used to train potentials for each system.[S9] Training protocols, neural network architectures for machine learning interatomic potentials (MLIPs), and the loss function used are identical to our previous work.[S10] We used the `se_e2_a` descriptor, which uses both angular and radial information, and uses embedding that contains two-atom information. We set a cutoff radius of 6 Å with a smoothing cutoff of 2 Å. Training for each round of active learning (AL) was performed for 1 million batches for the reactive active learning (RAL) cycles. However, the SC-AL training used 200,000 batches in each subcycle. The embedding network consisted of three hidden layers with 25, 50, and 100 neurons. The

fitting network used a cylindrical layout with three hidden layer with each containing 240 neurons. These training settings were consistent for all test cases.

Table S1: Chemical formulas and SMILES representations of reactants and products used for ammonia synthesis and methanimine hydrolysis reactions.

Chemical formula	SMILES string
N_2	N#N
H_2	[H][H]
NH_3	[NH3]
N_2H_4	NN
N_2H_2	N=N
${N_2H_2}^{2+}$	[NH+]#[NH+]
${N_2H_3}^{+}$	N=[NH2+]
H	[H]
CH_2NH	C=N
H_2O	[O]
CH_3NH_2	CN
CH_2NH_2OH	NCO
CH_2O	C=O
H_2	[H][H]
CH_3NHOH	CNO

S3 Transition State Finding Methods

A major part of automating the process of reaction exploration was performed using established transition state (TS) finding codes. The single-ended growing string method (SE-GSM)[S11] and the nudged elastic band (NEB)[S12] method are both techniques for locating probable minimum energy pathways (MEPs) and transition states in chemical reactions. SE-GSM works by incrementally adjusting the geometry of a reaction pathway starting from the reactant state and effectively “growing” the reaction string towards the product. SE-GSM only requires an initialization of the driving internal coordinates and the geometry of the reactant structure to propagate a reaction. Most TS-finding methods are limited to elementary reaction pathways, which makes SE-GSM advantageous because there is no constraint on the form of the product. Also, it makes SE-GSM particularly useful for exploring

complex reaction landscapes. We used pyGSM, a Python version of the original growing string method, which is compatible with the Atomic Simulation Environment (ASE). In this study, SE-GSM was only used for reaction exploration in the homogeneous phase. We used NEB for periodic systems since the version of pyGSM at the time of publication was only compatible with non-periodic systems. NEB connects reactants and products with a series of intermediate images (replicas of the system). The method applies forces that push the images up the potential energy surface and perpendicular forces to keep the images spaced evenly, allowing for the identification of the MEP and the TS, through the use of climbing image NEB, which we use in our work.[S13–S15]

S4 Evaluation metrics

We employed a suite of metrics designed to quantify the diversity and convergence of sampled molecular configurations to evaluate the performance of our training protocol. Firstly, we utilized the uniform manifold approximation and projection (UMAP)[S16] method to generate a two-dimensional representation of our high-dimensional molecular data, derived from Smooth Overlap of Atomic Positions (SOAP) descriptors.[S17] This visualization allowed us to assess the geometric dissimilarity between intermediate structures generated during RAL training, with spatial separation in the UMAP space indicating distinct geometries. Secondly, the convex hull volume of the UMAP distribution was calculated to quantify the diversity of sampled intermediates in each RAL generation; an increasing convex hull volume across successive generations signified effective exploration of configurational space. Finally, we implemented a cumulative nearest neighbor (NN) distance metric.[S18] Specifically, we fitted the NN model to the cumulative UMAP embedding of the preceding generations and subsequently predicted the distances for the nth generation. This analysis focused solely on the first NN, representing the closest point within the distribution. The NN of any given point indicates its most structurally similar counterpart. The NN distance between the nth

generation and the cumulative $n-1$ generations was determined by calculating the distances for each point in the current generation with respect to the points in the sum of all previous generations. We anticipated that the cumulative NN distance would decrease with successive generations, signifying a lower diversity in the configurations being explored as n increases. This indicates that generations with small values of the cumulative NN distance are less efficient than those with large values, because most of the diverse structures available have already been explored. Thus, in the cumulative NN distance should tend to zero as the number of generations increases.

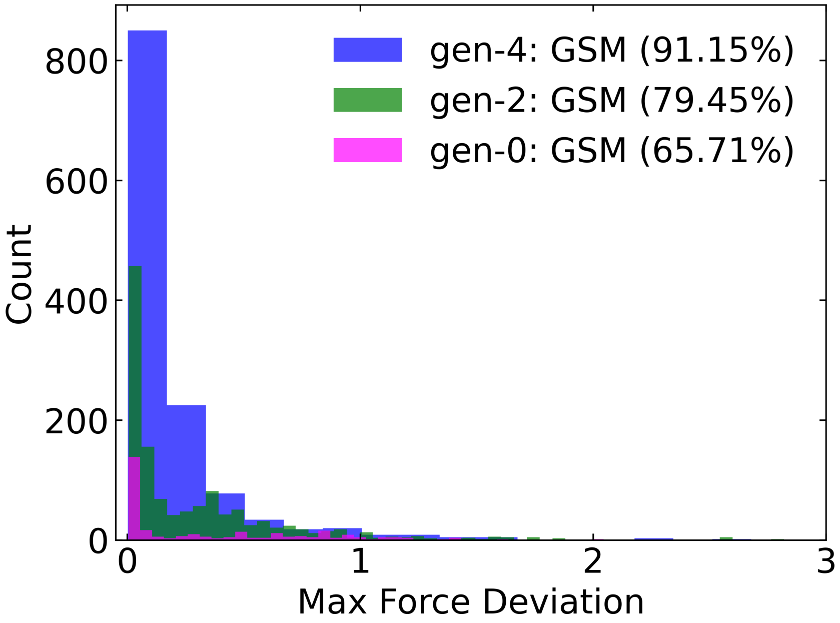

Figure S1: Histogram of maximum force deviations for SE-GSM images for the ammonia system using the nonself-consistent-AL (NSC-AL) approach, shown for gen-0 (magenta), gen-2 (green), and gen-4 (blue). The parentheses include the percentage of images with maximum force deviations less than 0.5 eV/Å.

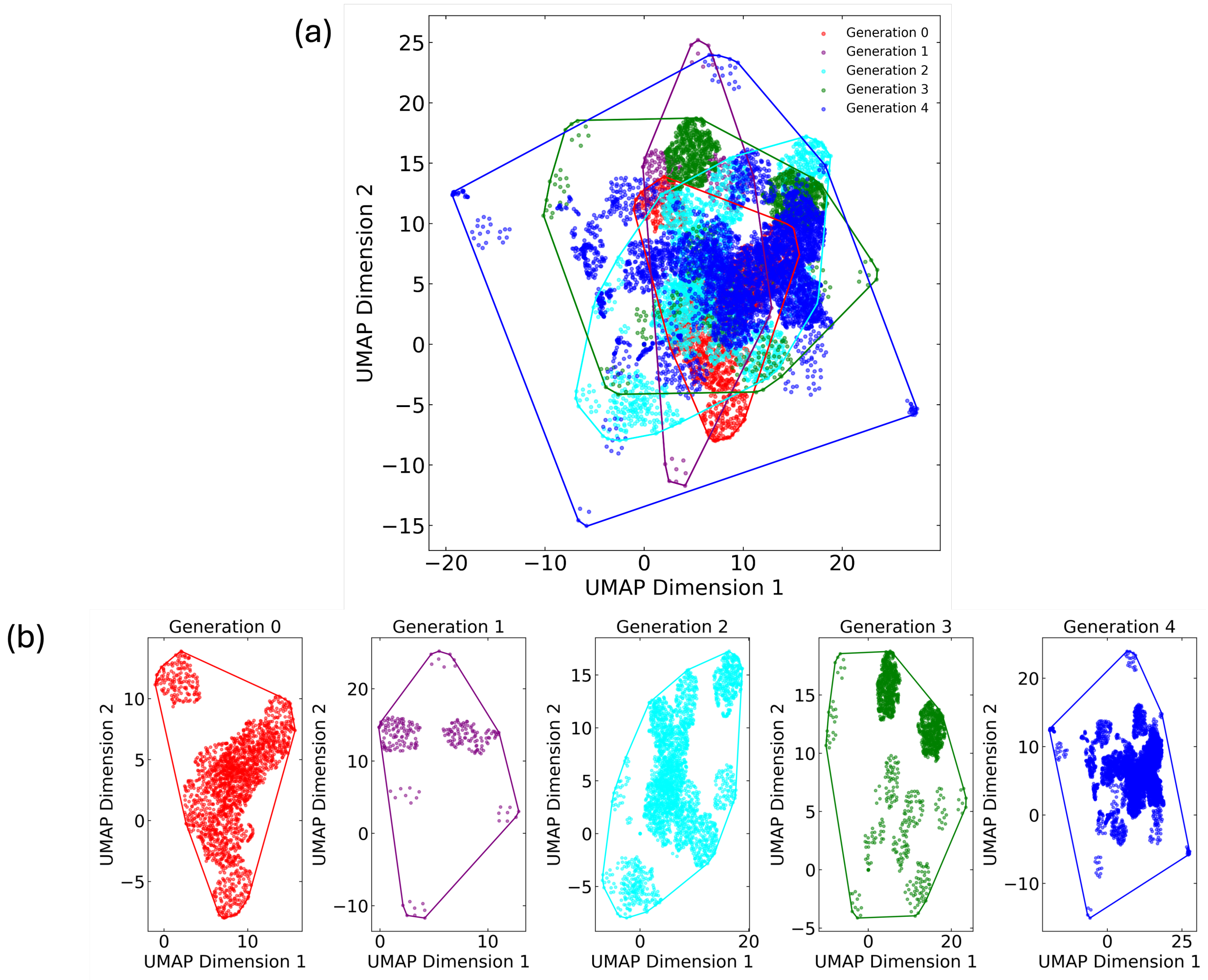

Figure S2: (a) Overlapped and (b) individual UMAP plots for the ammonia system using the NSC-AL approach, shown for gen-0 (red), gen-1 (purple), gen-2 (cyan), gen-3 (green), and gen-4 (blue).

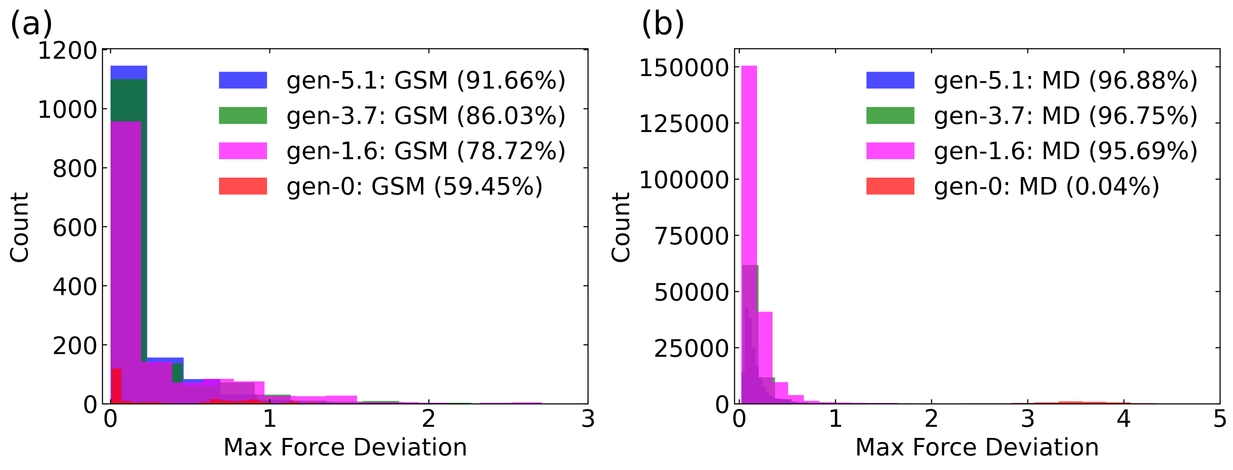

Figure S3: Histogram of maximum force deviations for (a) SE-GSM and (b) MD images for the ammonia system using the self-consistent-AL (SC-AL) approach, shown for gen-0 (red), gen-1.6 (magenta), gen-3.7 (green), and gen-5.1 (blue), respectively. The parentheses include the percentage of images with maximum force deviations less than 0.5 eV/Å.

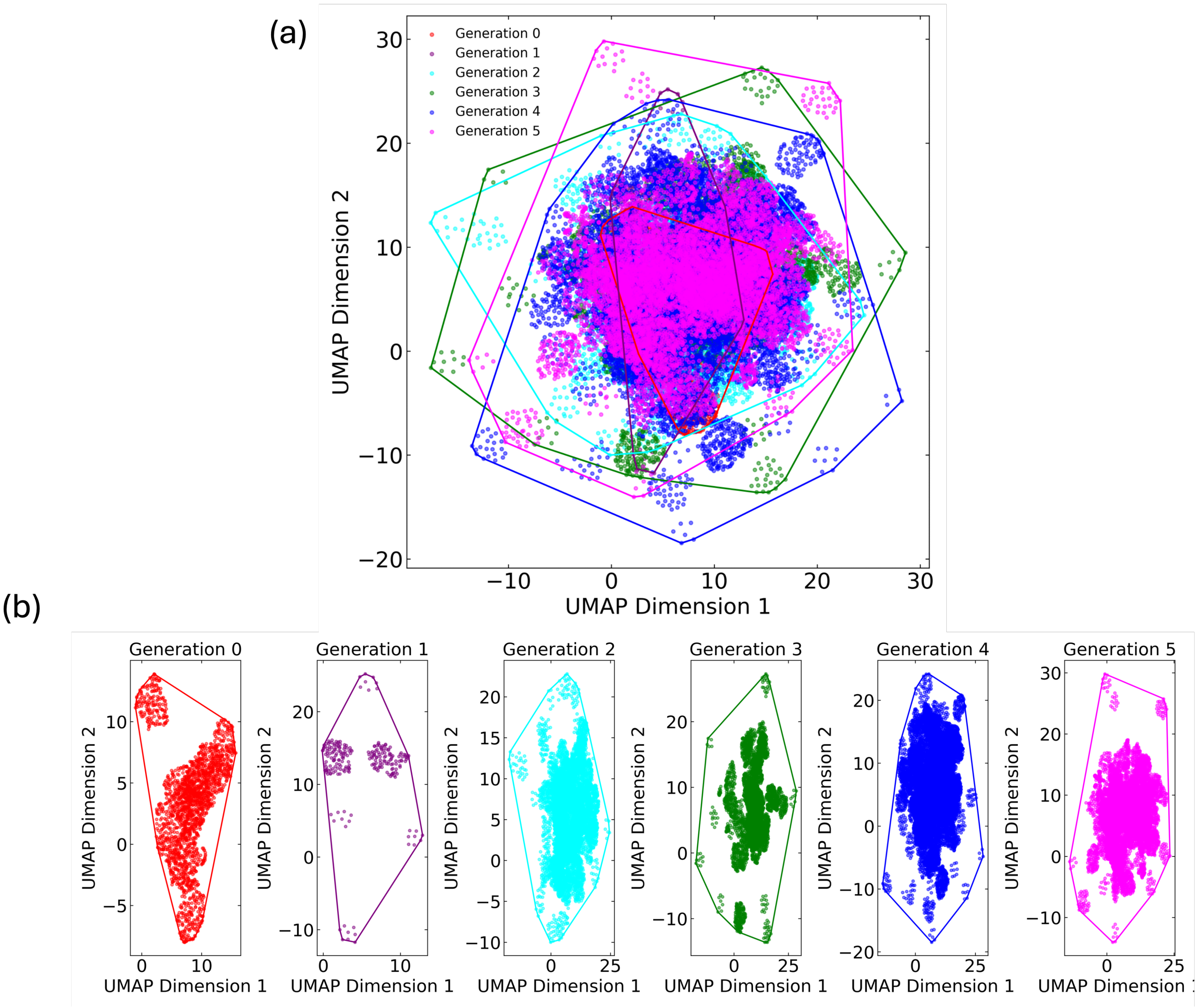

Figure S4: (a) Overlapped and (b) individual UMAP plots for the ammonia system using the SC-AL approach, shown for gen-0 (red), gen-1 (purple), gen-2 (cyan), and gen-3 (green), gen-4 (blue), and gen-5 (magenta).

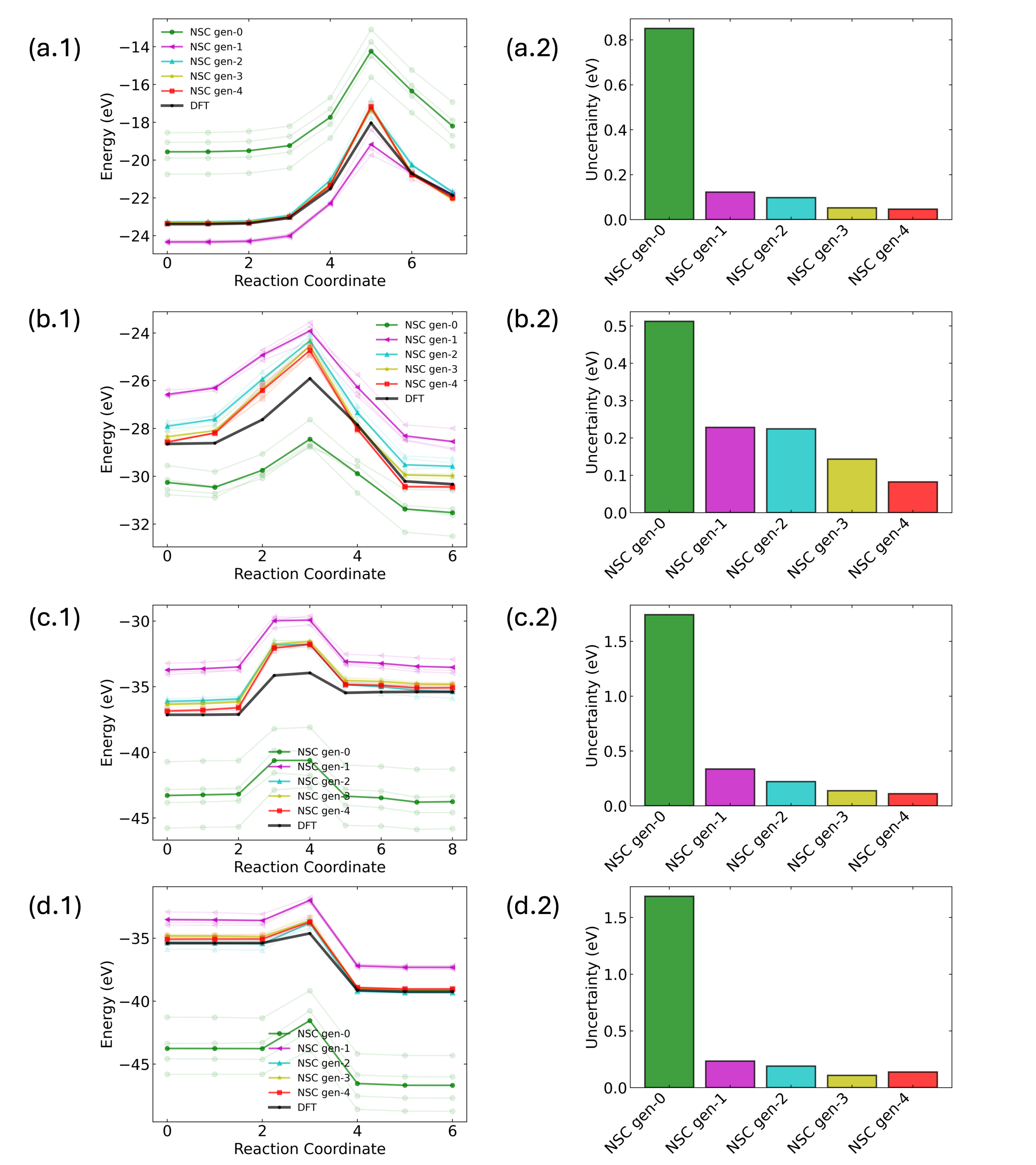

Figure S5: (a.1–d.1) Predicted energy profiles along the reaction coordinate for four benchmark reactions in the ammonia system, corresponding to Figure 4 in the main text. Results from NSC-AL gen-0 (green, circles), gen-1 (magenta, left triangles), gen-2 (cyan, up triangles), gen-3 (yellow, stars), and gen-4 (red, squares) are shown alongside benchmark DFT calculations (black, small circles). The mean predicted MLIP energy is plotted in bold for each generation, while predicted energies from the ensemble of MLIPs are plotted in the same color with increased transparency. (a.2-d.2) Uncertainty estimates for each reaction across the four NSC-AL generations. All energies are reported in eV.

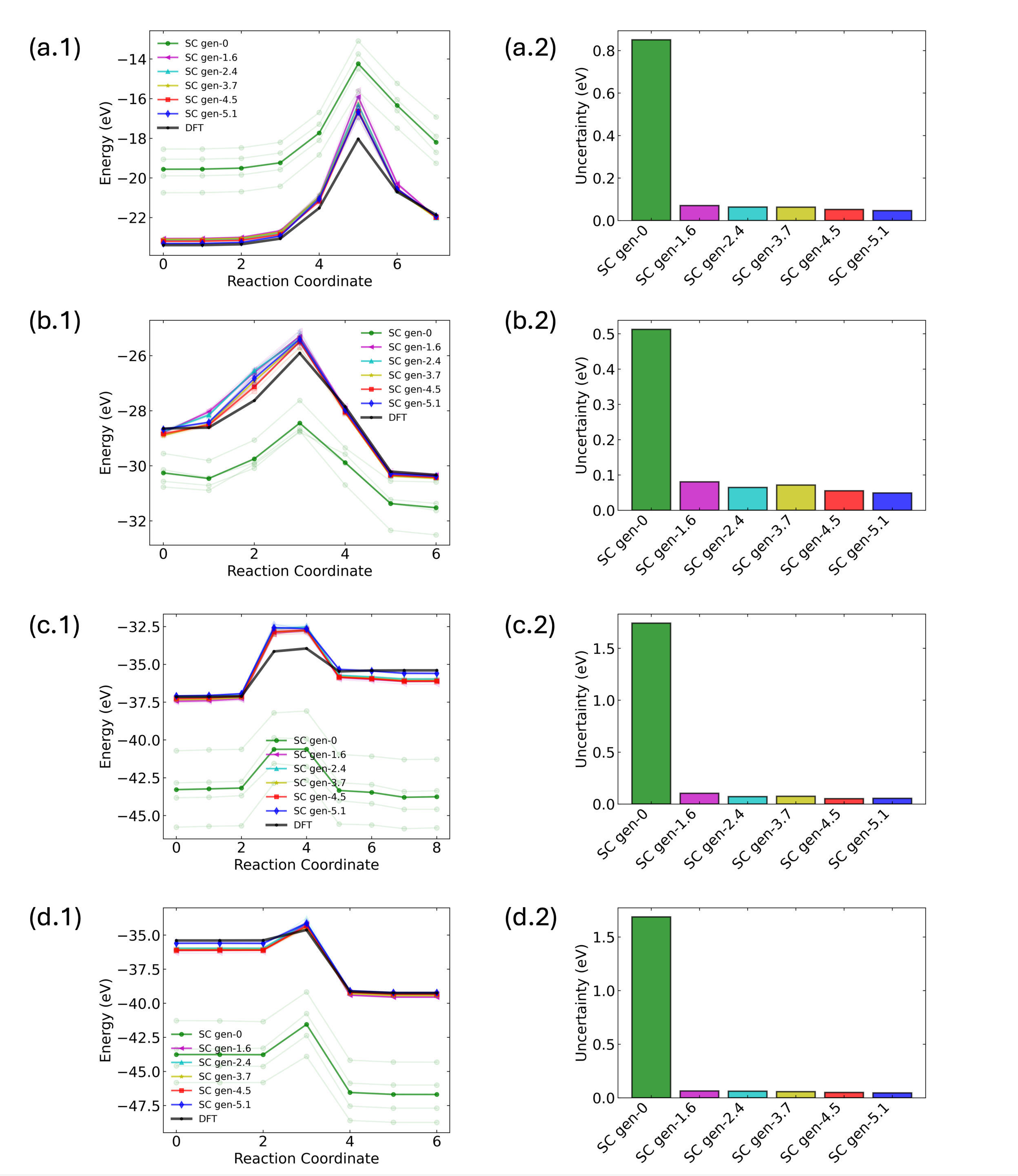

Figure S6: (a.1-d.1) Predicted energy profiles along the reaction coordinate for four benchmark reactions in the ammonia system, corresponding to Figure 4 in the main text. Results from SC-AL gen-0 (green, circles), gen-1.6 (magenta, left triangles), gen-2.4 (cyan, up triangles), gen-3.7 (yellow, stars), gen-4.5 (red, squares), and gen-5.1 (blue, diamonds) are shown alongside benchmark DFT calculations (black, small circles). The mean predicted MLIP energy is plotted in bold, while predicted energies from each of the MLIPs are plotted in the same color with increased transparency. (a.2-d.2) Uncertainty estimates for each reaction across the five SC-AL generations. All energies are reported in eV.

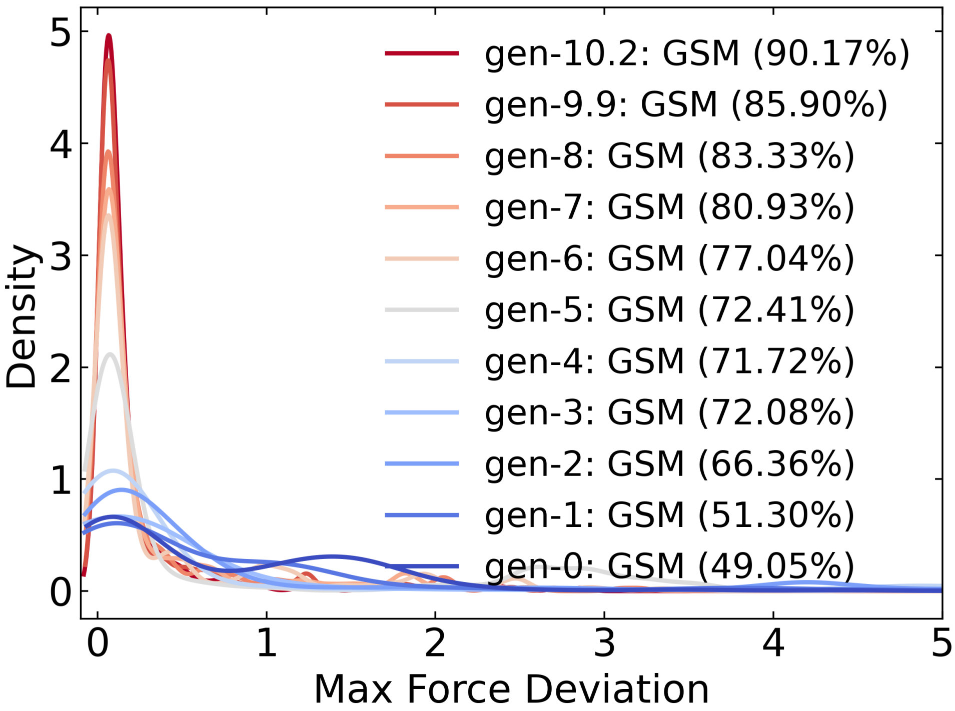

Figure S7: Density plot of maximum force deviations for SE-GSM images for the methanimine-water system using the hybrid-AL approach, shown for gen-0 through gen-8, gen-9.9, and gen-10.2. The parentheses include the percentage of images with maximum force deviations less than 0.5 eV/Å.

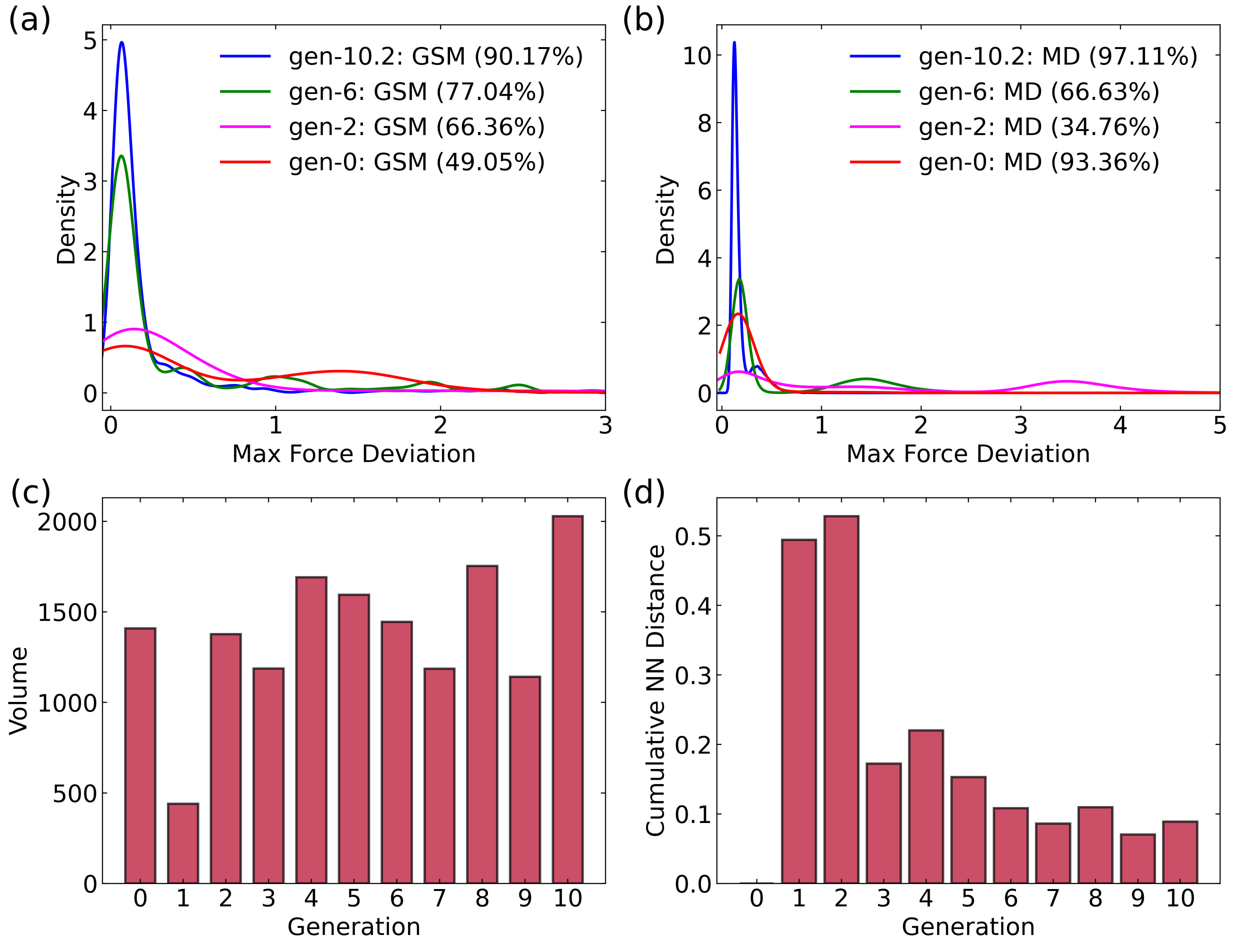

Figure S8: Maximum force deviations for (a) SE-GSM and (b) MD images for the methanimine-water system with the hybrid-AL approach, shown for gen-0 (red), gen-2 (magenta), gen-6 (green), and gen-10.2 (blue), respectively. The parentheses include the percentage of images with maximum force deviations less than 0.5 eV/Å. (c) Convex hull volume, shown for gen-0 to gen-10. (d) Cumulative NN distance between consecutive generations from gen-0 to gen-10.

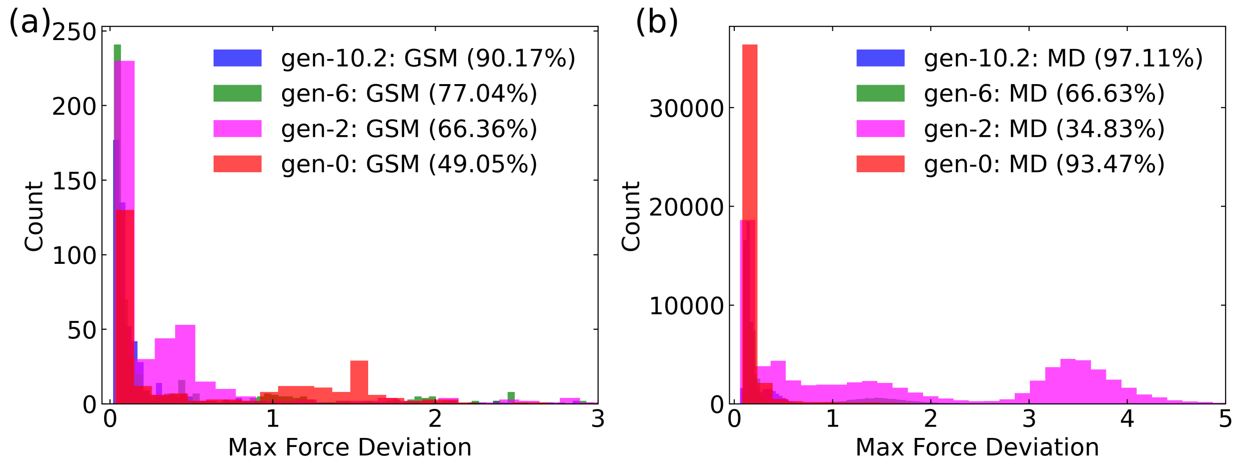

Figure S9: Histograms of maximum force deviations for (a) SE-GSM and (b) MD images for the methanimine-water system using the hybrid-AL approach, shown for gen-0 (red), gen-2 (magenta), gen-6 (green), and gen-10.2 (blue), respectively. The parentheses include the percentage of images with maximum force deviations less than 0.5 eV/Å.

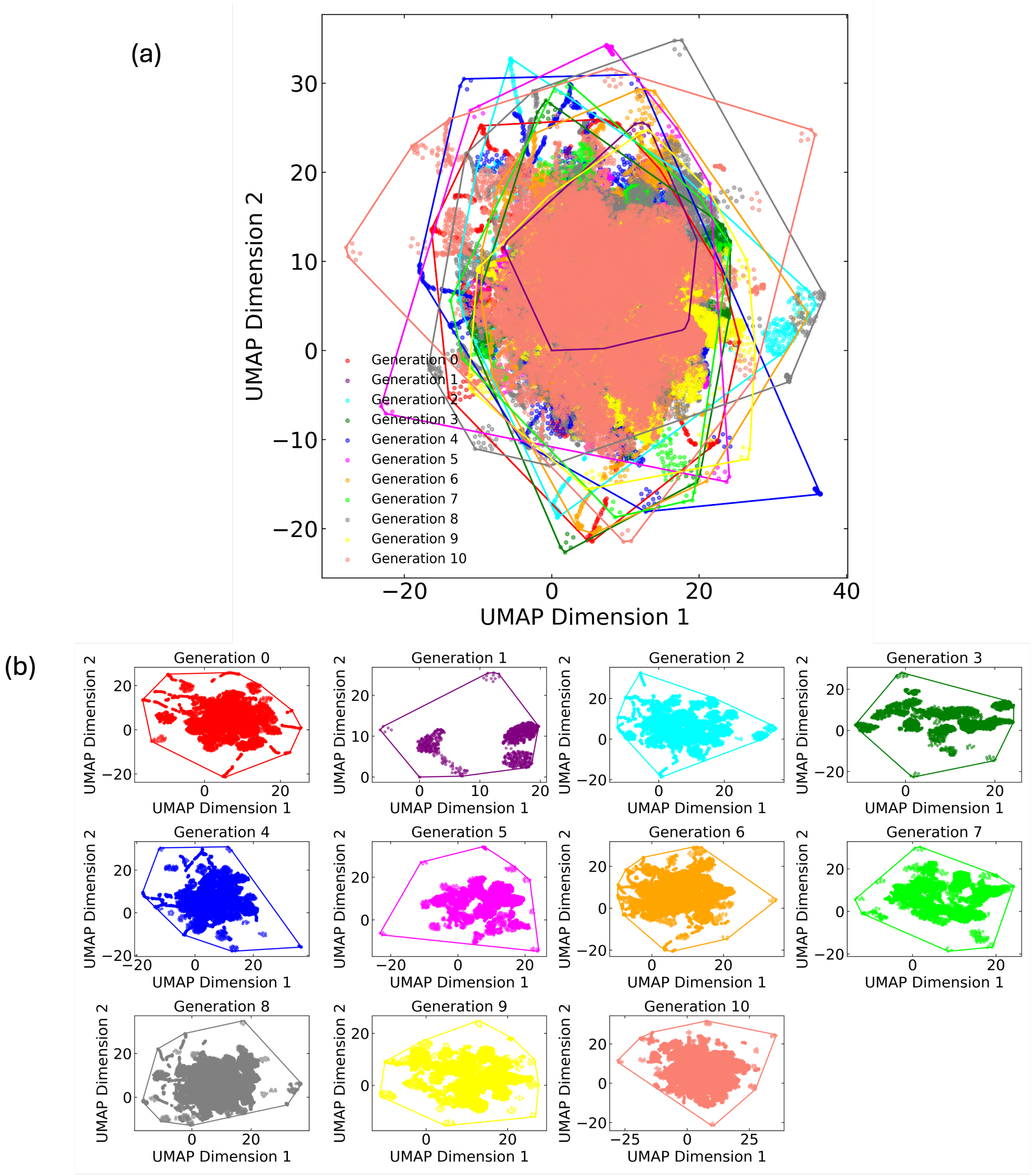

Figure S10: (a) Overlapped and (b) individual UMAP plots for the methanimine-water system with the hybrid-AL approach, shown for gen-0 (red), gen-1 (purple), gen-2 (cyan), gen-3 (green), gen-4 (blue), gen-5 (magenta), gen-6 (orange), gen-7 (lime), gen-8 (gray), gen-9 (yellow), and gen-10 (salmon).

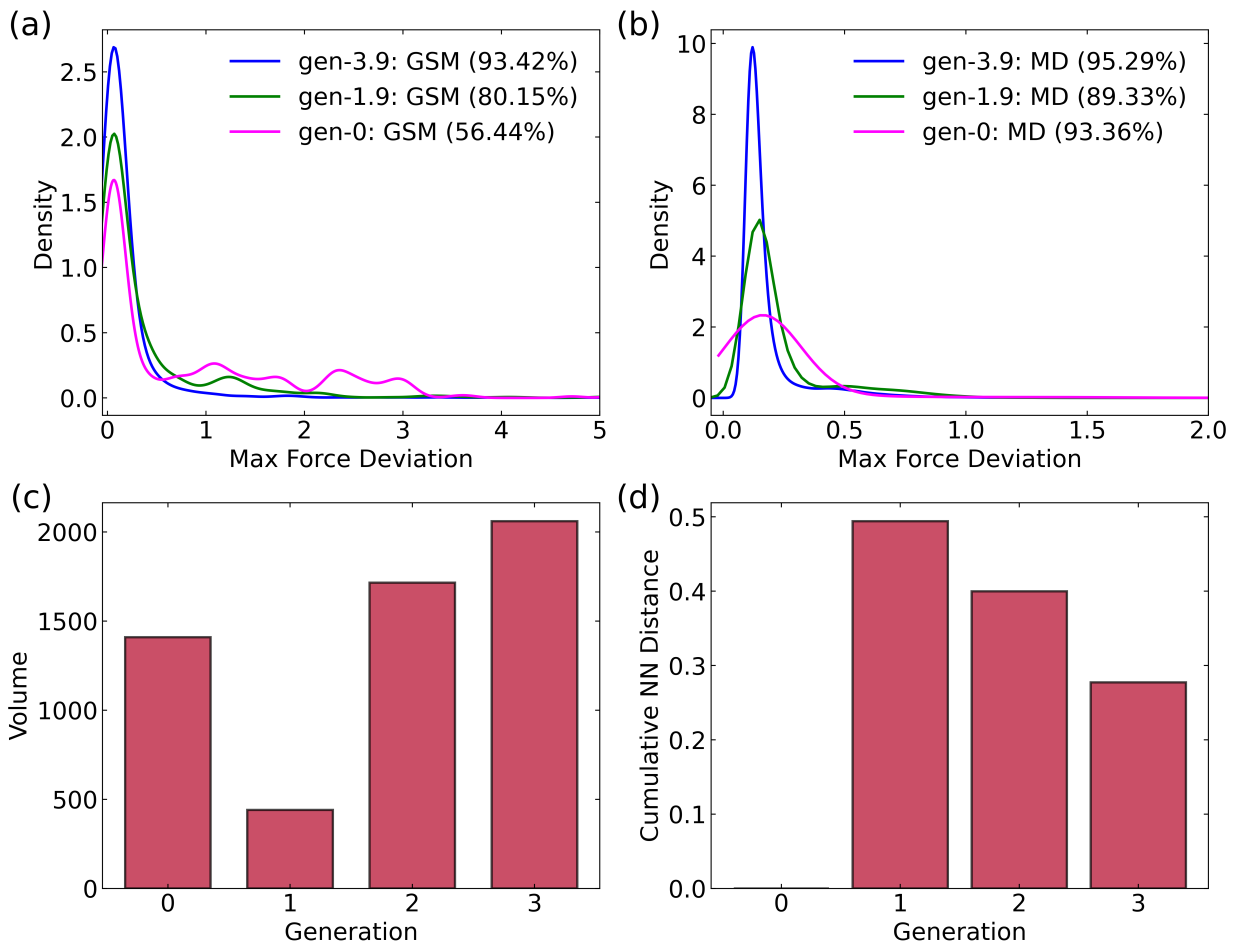

Figure S11: Maximum force deviations for (a) SE-GSM and (b) MD images for the methanimine-water system using the SC-AL approach, shown for gen-0 (magenta), gen-1.9 (green), and gen-3.9 (blue), respectively. The parentheses include the percentage of images with maximum force deviations less than 0.5 eV/Å. (c) convex hull volume, shown for gen-0 to gen-3, and (d) cumulative NN distance between consecutive generations from gen-0 to gen-3.

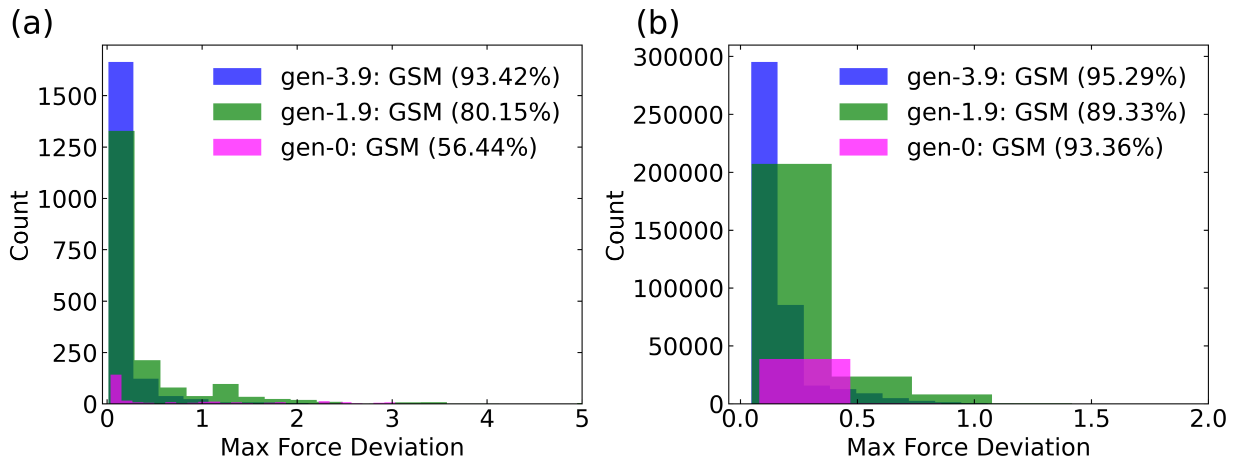

Figure S12: Histogram of maximum force deviations for (a) SE-GSM and (b) MD images for the methanimine-water system using the SC-AL approach, shown for gen-0 (magenta), gen-1.9 (green), and gen-3.9 (blue), respectively. The parentheses include the percentage of images with maximum force deviations less than 0.5 eV/Å.

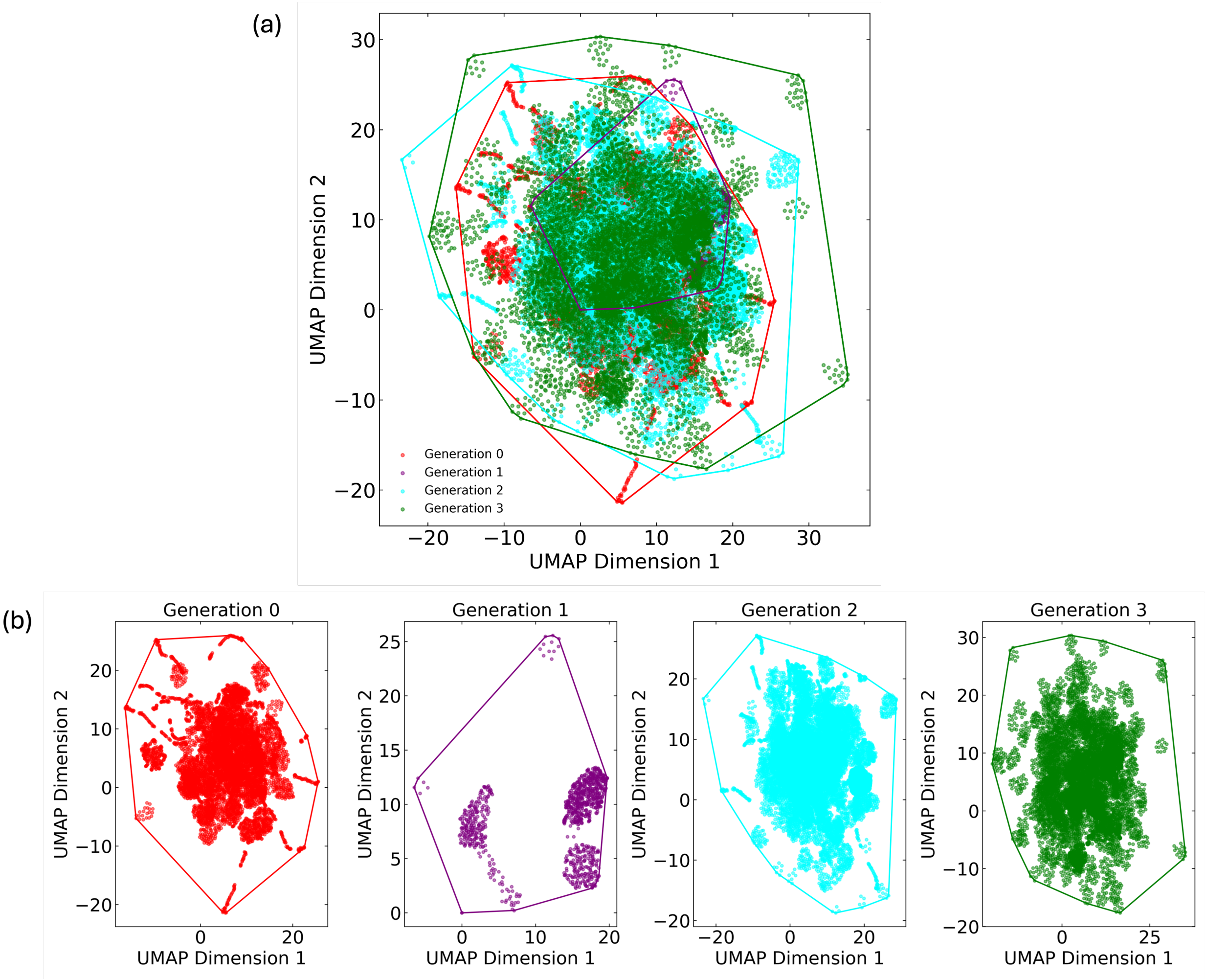

Figure S13: (a) Overlapped and (b) individual UMAP plots for the methanimine-water system using the SC-AL approach, shown for gen-0 (red), gen-1 (purple), gen-2 (cyan), and gen-3 (green).

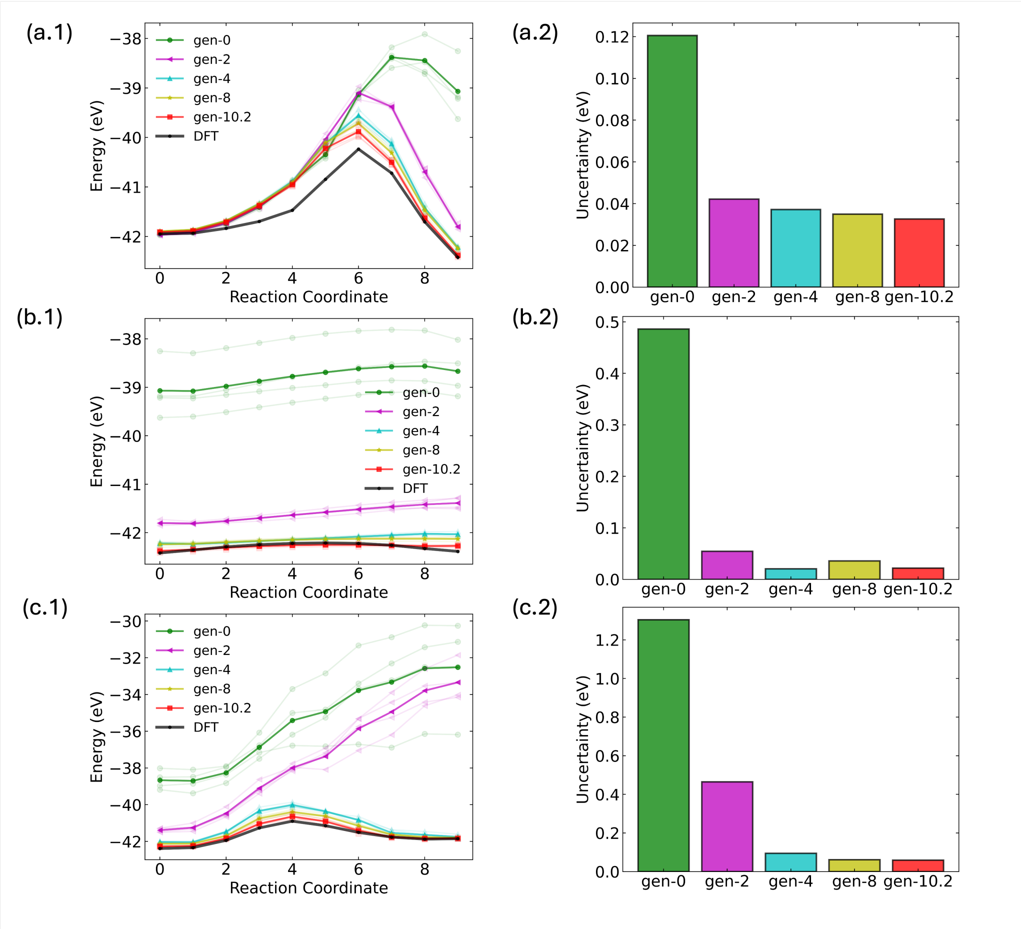

Figure S14: (a.1-c.1) Predicted energy profiles along the reaction coordinate for three benchmark reactions in the methanimine-water system, corresponding to Figure 5 in the main text. Results from hybrid-AL gen-0 (green, circles), gen-2 (magenta, left triangles), gen-4 (cyan, up triangles),gen-8 (yellow, stars), and gen-10.2 (red, squares) are shown alongside benchmark DFT calculations (black, small circles). The mean predicted MLIP energy is plotted in bold, while predicted energies from the ensemble of MLIPs are plotted in the same color with increased transparency. (a.2-c.2) Uncertainty estimates for each reaction across the hybrid-AL generations. All energies are reported in eV.

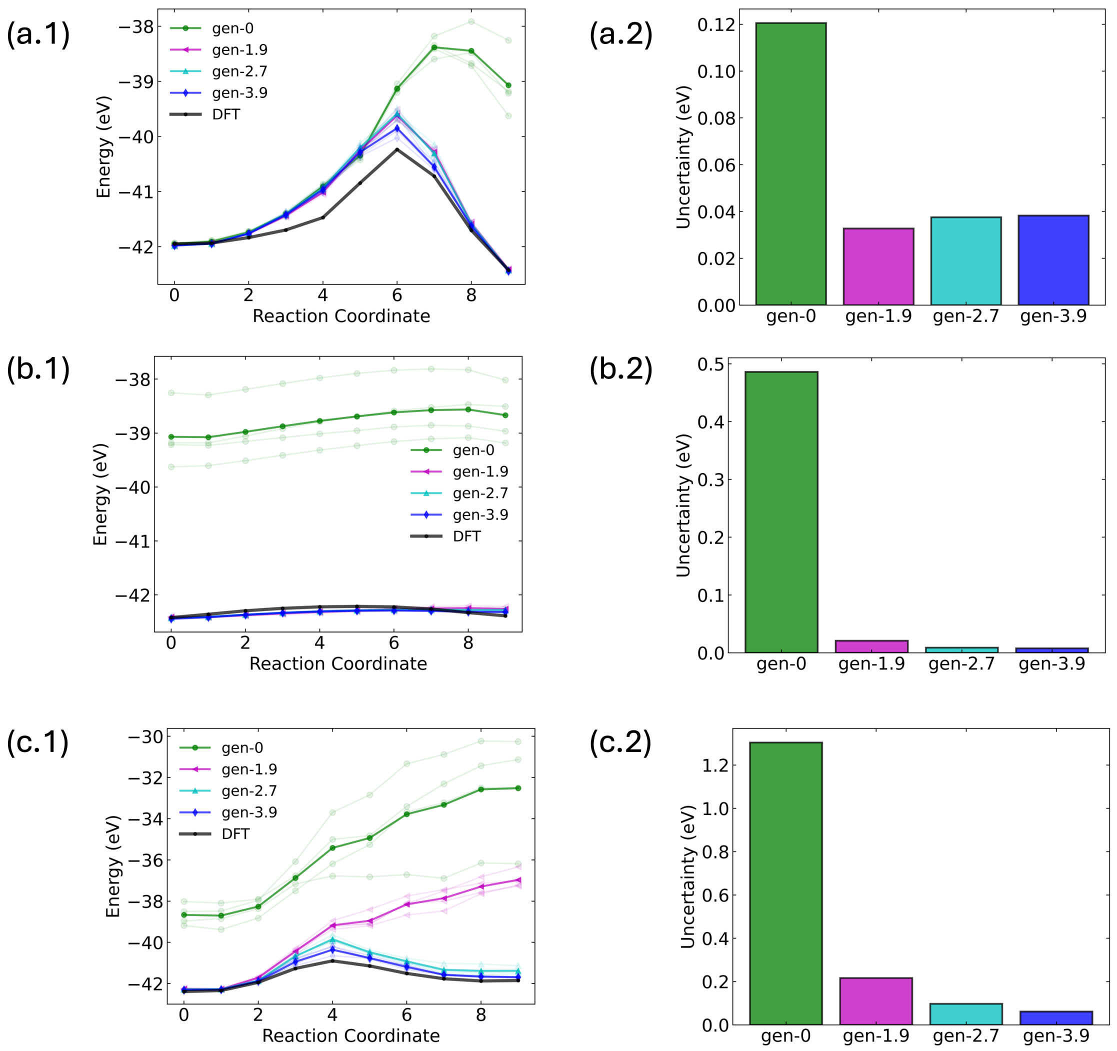

Figure S15: (a.1-c.1) Predicted energy profiles along the reaction coordinate for three benchmark reactions in the methanimine-water system, corresponding to Figure 5 in the main text. Results from SC-AL gen-0 (green, circles), gen-1.9 (magenta, left triangles), gen-2.7 (cyan, up triangles), and gen-3.9 (blue, diamonds) are shown alongside benchmark DFT calculations (black, small circles). The mean predicted MLIP energy is plotted in bold, while predicted energies from the ensemble of MLIPs are plotted in the same color with increased transparency. (a.2-c.2) Uncertainty estimates for each reaction across the SC-AL generations. All energies are reported in eV.

S5 Nanoreactor Method

Zhang *et al.*[S19] implemented a nanoreactor (NR) approach to perform AL sampling for reactive systems composed of C, H, N, and O. The NR simulations of Zhang *et al.* were initialized with random compositions of small molecules (H_2, N_2, O_2, NH_3, CH_4, CO_2, H_2O, and C_2H_2). They used initial temperatures and initial densities randomly chosen between 1000–3000 K and 0.1–2 g/mL. The ranges for final temperature and final density were between 100–2000 K and 0.5–2 g/mL. To encourage chemical reactions, Zhang *et al.* applied large fluctuations in the temperature ($\leq$ 2000 K) and large fluctuations in the density ($\leq$ 0.75 g/mL), causing extremely high instantaneous pressures. They used the Accurate NeurAl networK engINe for Molecular Energies (ANI)[S20] neural network architecture. The NR-AL approach provided multiple intermediates without intensive QM sampling. Through multiple iterations of AL, the extended ANI-1x-nr dataset covered chemical space.

We compared the NR-AL approach to the RAL method for the methanimine hydrolysis reaction. We note that it is not clear how to apply the NR-AL method to heterogeneous systems like our Ti_xC_y catalysis system. To begin the NR-AL protocol, we used the same initial systems used to train the bootstrap potential methanimine hydrolysis reaction, in order to maintain consistency between RAL and the NR-AL approach. We generated 29 NR-AL generations using these initial densities, but found limited improvement in the quality of the MLIPs. We determined that many of the densities explored in these generations were too low compared with densities typically sampled in the NR-AL approach, because high densities are required to effectively sample chemical reactions. We therefore generated 34 high-density systems, with densities ranging from 1.2 to 1.8 g/ml, and used these as the starting configurations for additional generations of NR-AL training. These systems were used to produce gen-30 to gen-47 of the NR MLIPs. We only show results generations after gen-30, as the AL cycles after gen-30 contain structures in a similar density range explored by Zhang et al.[S19] We varied the temperature and volume of the simulation cell using a

sinusoidal temporal function similar to Zhang *et al.*,[S19] as given in Equations (S1) and (S2):

$$T(t) = T_{\text{start}} + \frac{t}{t_{\text{max}}}(T_{\text{end}} - T_{\text{start}}) + T_{\text{amp}} \sin^2(\omega_T t) \tag{S1}$$

$$\rho(t) = \rho_{\text{start}} + \frac{t}{t_{\text{max}}}(\rho_{\text{end}} - \rho_{\text{start}}) + \rho_{\text{amp}} \sin^2(\omega_\rho t) \tag{S2}$$

where, $\omega_i = \frac{2\pi}{t_{\text{per},i}}$, with $i = T$ or ρ, $t_{\text{per},i}$ is the period of variation for either the temperature in Eq. (S1) or the density in Eq. (S2), ρ_{start} is the lowest possible value in the variation range of the quantity, ρ_{end} is the highest possible value in the variation range of the quantity, ρ_{amp} is the amplitude of the variation, and t_{max} is the maximum simulation run time. Nanoreactor molecular dynamics (MD) simulations were run using the Atomistic Simulation Environment (ASE)[S21] MD library using the Andersen thermostat.[S22] The ranges of hyperparameters for methanimine system are listed in Table S2.

Table S2: Range of hyperparameters used in the sinusoidal variation functions of Eqns (S1) and (S2)

T_{start} (K)	800–1000
T_{end} (K)	1000–2000
T_{amp} (K)	100–500
$t_{\text{per},T}$ (ps)	2–50
ρ_{start} (g/cc)	0.15–1.8
ρ_{end} (g/cc)	1.8–2.5
ρ_{amp} (g/cc)	0.04–0.2
$t_{\text{per},\rho}$ (ps)	2–50

The NR-AL method uses energy and force thresholds to determine when to terminate a specific NR-AL exploration simulation. These were checked at every iteration. The final value of force cutoff and the energy cutoff were 1.1 eV/Åand 0.6 eV/$N^{1/2}$, respectively, where N is the number of atoms in the system. If the MD run exceeded either threshold, the final configuration was used for relabeling. The AL iterations are supposed to continue until the final convergence criterion was met, which is defined as almost all of the NR-MD simulations running for 50 ps or more. We performed 47 cycles of NR-AL, but the convergence criterion was not met, since only one of the NR-MD runs reached 50 ps. We did not run NR-AL to

convergence because by the time we reached 47 NR-AL cycles, we had effectively exceeded or matched the total computational effort, as measured in service units (SUs), required to train MLIPs with RAL, according to our best estimate. The SUs required for RAL and NR-AL training are given in Table S3, where the definition of an SU is also given. The SUs required for NR-AL training for gen-1–29 is 119169, which exceeds the SUs for either RAL training approach (see Table S3). However, to make a fair comparison we scaled this value by 0.4, since we estimated only about 40% of the configurations in these generations were in the higher density range typically sampled in the NR-AL approach. We also reduced the computational cost of training for gen-30–47 by reducing the number of batches from 1 million to 500,000. We therefore estimate the effective SU cost for the NR-AL approach for the methanimine system to be (0.4)119169 + 41457 = 89125 SUs. This value exceeds the SUs for the SC-AL RAL training and is slightly lower than the hybrid-AL approach (see Table S3).

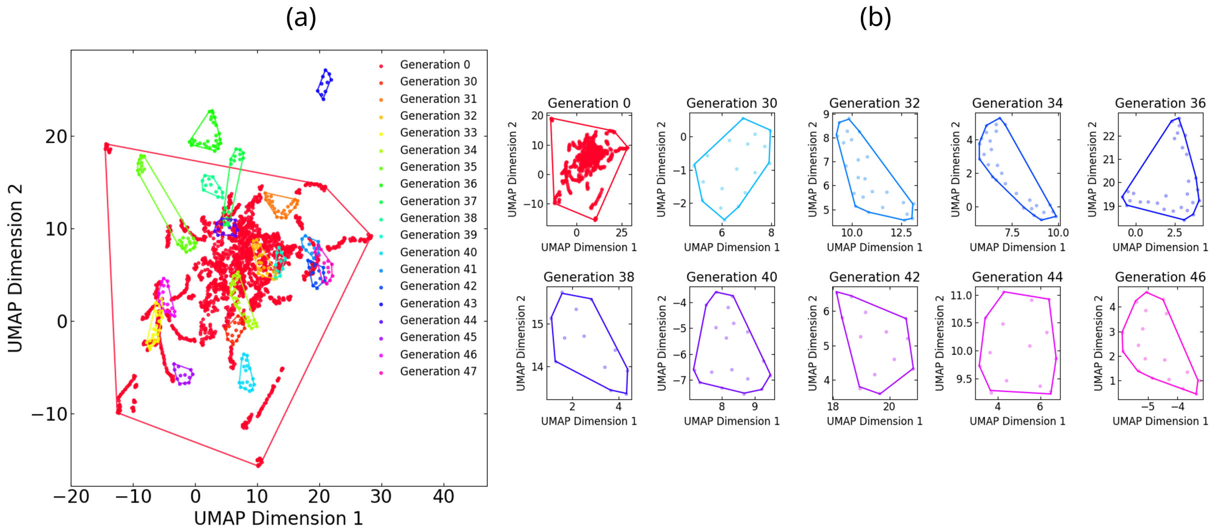

Figure S16: (a) Overlapped and (b) individual UMAP plots for the methanimine-water system using the NR-AL approach, shown for selected generations with gen-0 shown in red. The NR-AL training generated a few data points each generation, with a limited spread compared to gen-0. Multiple generations have configurations overlapping with gen-0 configurations, i.e., NR-AL failed to generate new configurations during training.

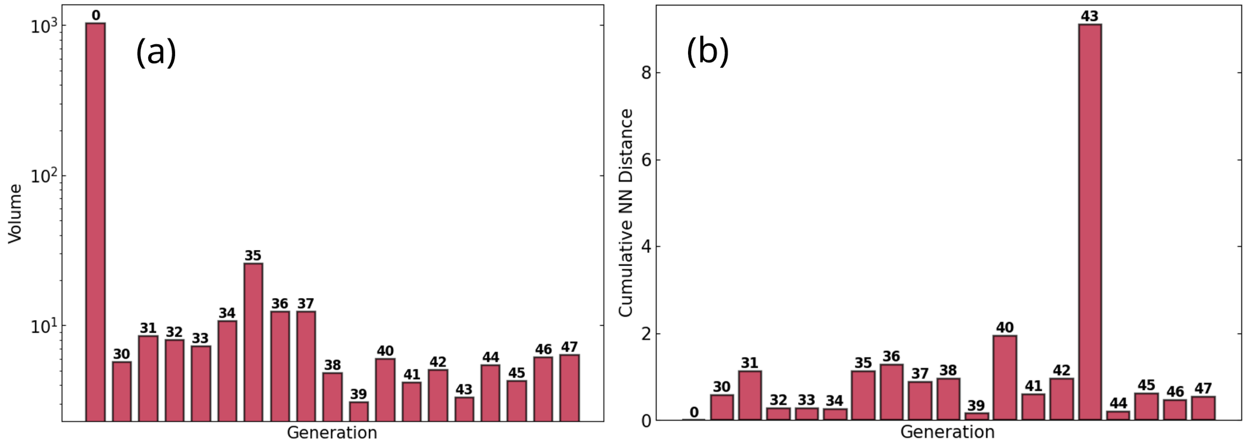

Figure S17: (a) Convex hull volumes for the methanimine-water system for 47 generations of NR-AL training; (b) The cumulative NN values for all generations of the methanimine-water system, respectively.

Table S3: Service units (SU) consumption for the (i) ammonia system using NSC-AL and SC-AL approaches; (ii) methanimine-water system using SC-AL, hybrid-AL and NR-AL approaches, (iii) Ti_xC_y system using the NSC-AL approach. The homogeneous systems used SE-GSM, and Ti_x C_y system used NEB for exploration. A major contribution to the SU consumption was from relabel-MD calculations. The SU is defined as one CPU core-hour of computing. SUs were calculated calculated for each calculation based on Trackable RESources (TRES) factors. TRES were calculated based on the number of cores requested, the RAM requested, the GPU cluster, and the number of cards requested.

	SU				
System	Train	Exploration	Relabel-GSM/NEB	Relabel-MD	Total
Ammonia (NSC-AL)	2127	90	22	4381	6620
Ammonia (SC-AL)	12218	370	378	37420	50386
Methanimine (SC-AL)	25047	388	1361	40372	67168
Methanimine (hybrid-AL)	41415	1001	965	49563	92944
Methanimine (NR-AL) gen-1–29	113054	4324	-	1791	119169
Methanimine (NR-AL) gen-30–47	34007	6200	-	1250	41457
Ti_xC_y	5661	274	18389	14239	38563

For GPU usage, a scaling factor of 8 was used to match the CPU compute resource. All GPU calculations were run with an Nvidia A100 GPU with AMD EPYC 7742 host architecture. All CPU calculations were run with AMD EPYC 9374F; this architecture defines the SU unit.

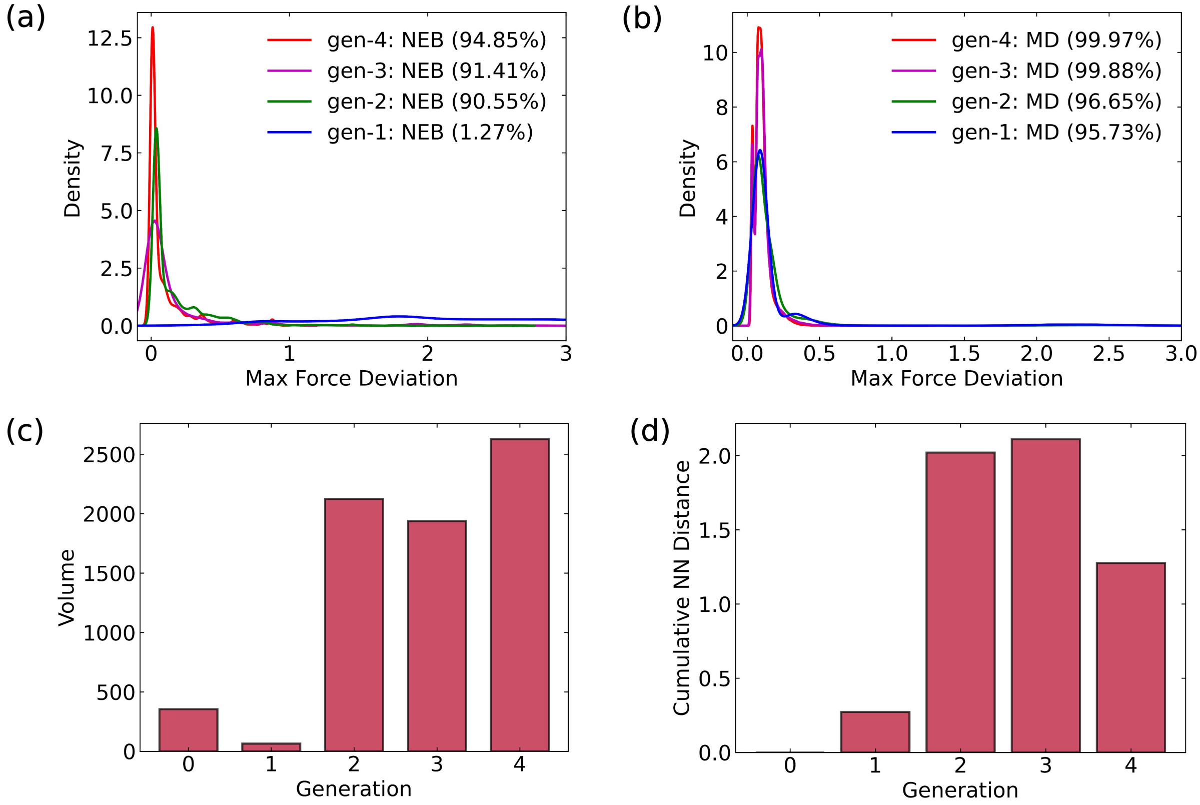

Figure S18: Maximum force deviations for (a) NEB and (b) MD images for Ti_xC_y heterogenous catalysis system using the NSC-AL approach, shown for gen-1 (blue), gen-2 (green), gen-3 (magenta), and gen-4 (red), respectively. The parentheses include the percentage of images with maximum force deviations less than 0.5 eV/Å. (c) convex hull volume, shown for gen-0 to gen-4, and (d) cumulative NN distance between consecutive generations from gen-0 to gen-4.

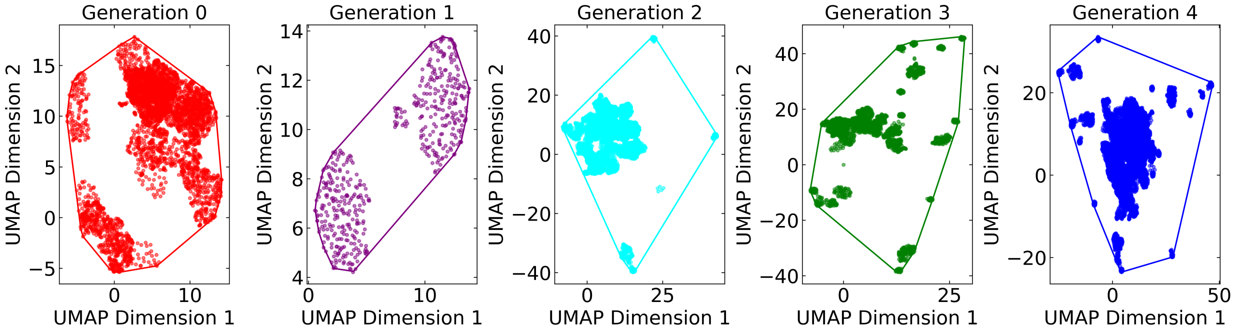

Figure S19: Individual UMAP plots for Ti_xC_y heterogenous catalysis system using the NSC-AL approach, shown for gen-0 (red), gen-1 (purple), gen-2 (cyan), gen-3, and gen-4 (green).

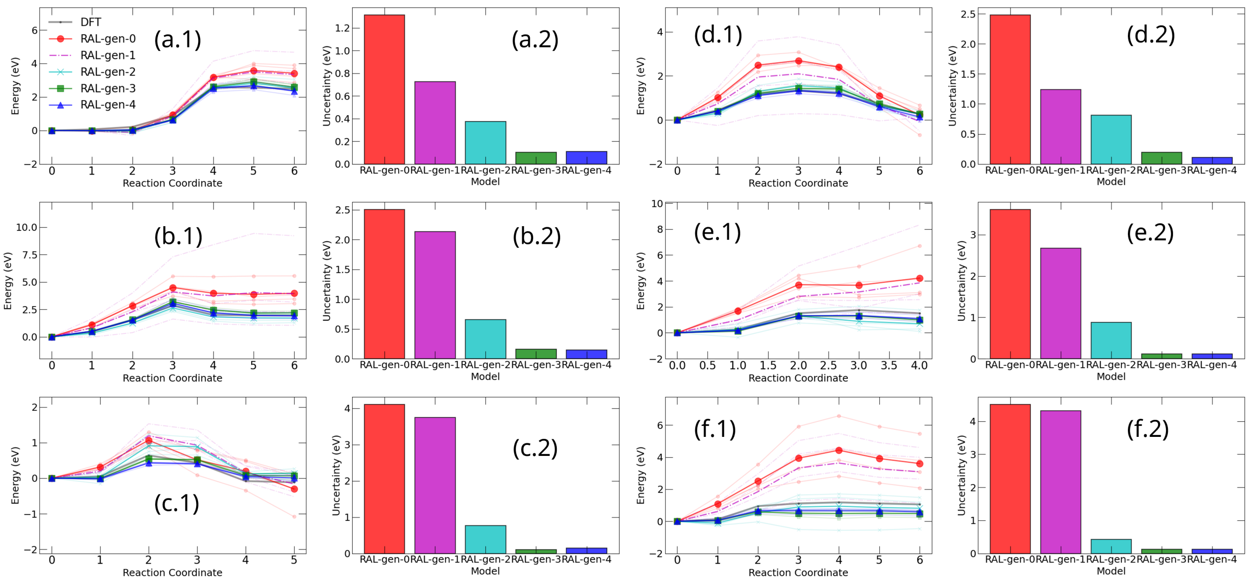

Figure S20: (a.1–f.1) Predicted energy profiles along the reaction coordinate for six heterogeneous TiC reaction systems for C-C coupling benchmark reactions, corresponding to Figure 6 in the main text. Results are shown for DFT (black, small circles), non-self-cosistent-AL (NSC-AL) gen-0 (red, circles), gen-1 (cyan), gen-2 (magenta, crosses), gen-3 (green, squares), and gen-4 (blue, triangles). The mean predicted MLIP energy is plotted in bold, while predicted energies from the ensemble of MLIPs are plotted in the same color with increased transparency. (a.2–f.2) Uncertainty estimates for each reaction across the NSC-AL generations. All energies are in eV.

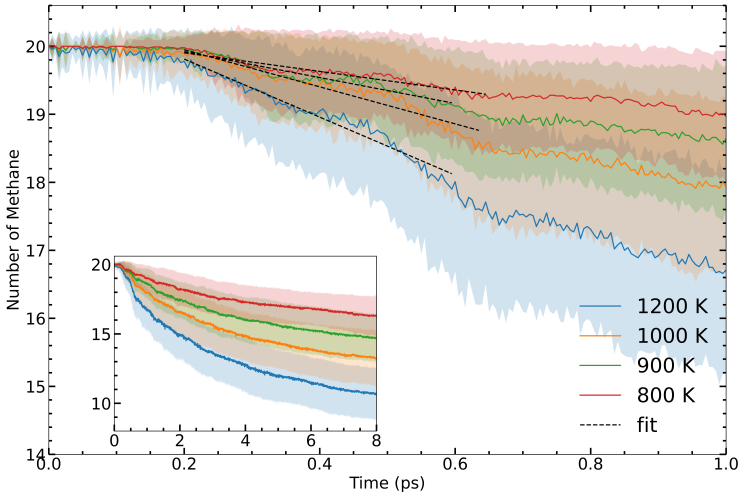

(a) $Ti_2C(100)$

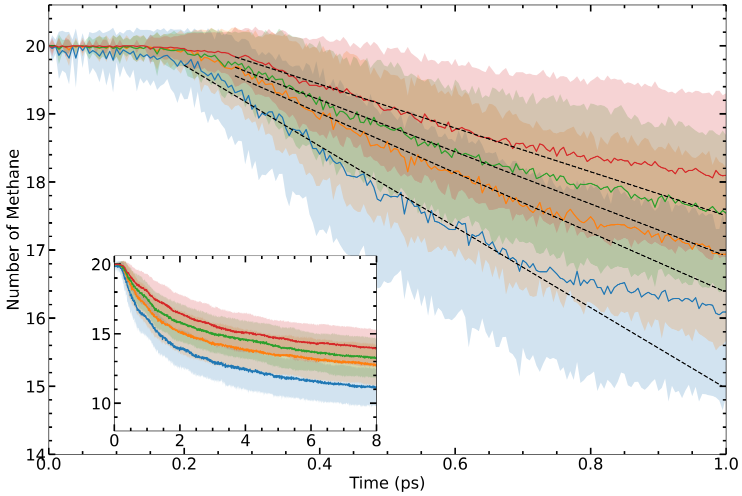

(b) $Ti_8C_5(001)$

Figure S21: Number of CH_4 molecules vs. time (in ps) for (a) Ti_2C and (b) Ti_8C_5 at four temperatures (1200, 1000, 900, and 800 K) averaged over 100 independent runs. The dashed lines are linear fits to the data.

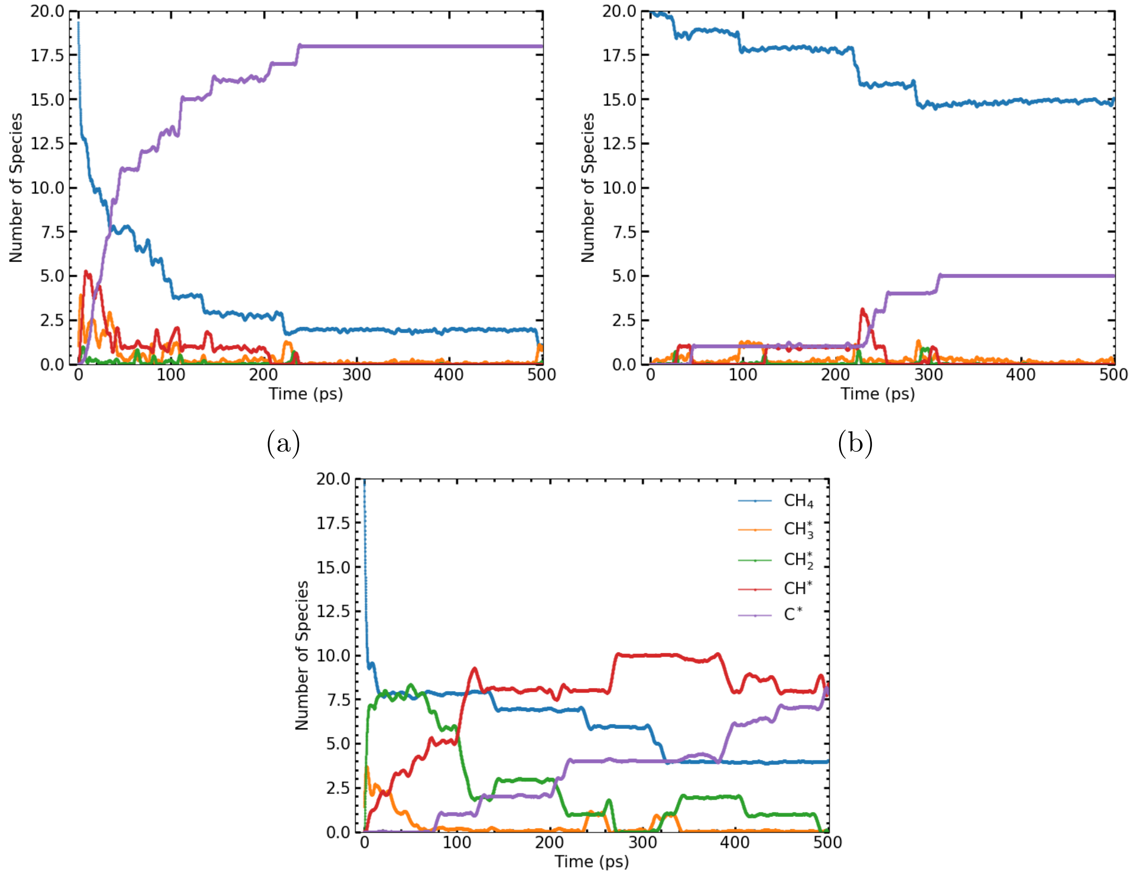

(c)

Figure S22: Evolution of the number of methane molecules and chemisorbed species on the $Ti_2C(100)$ surface after the (a) initial dose and (b) second dose. C* is the dominant adsorbed species at the end of the initial dose, indicating that most methane molecules convert to a single carbon atom on the surface of Ti_2C. The reactivity decreases at the end of the first dose, with hardly any methane in the system converting to intermediates. (c) Product distribution on Ti_8C_5 for the initial dose. There are no dominant products in Ti_8C_5, instead both C* and CH* are generated in significant proportions.

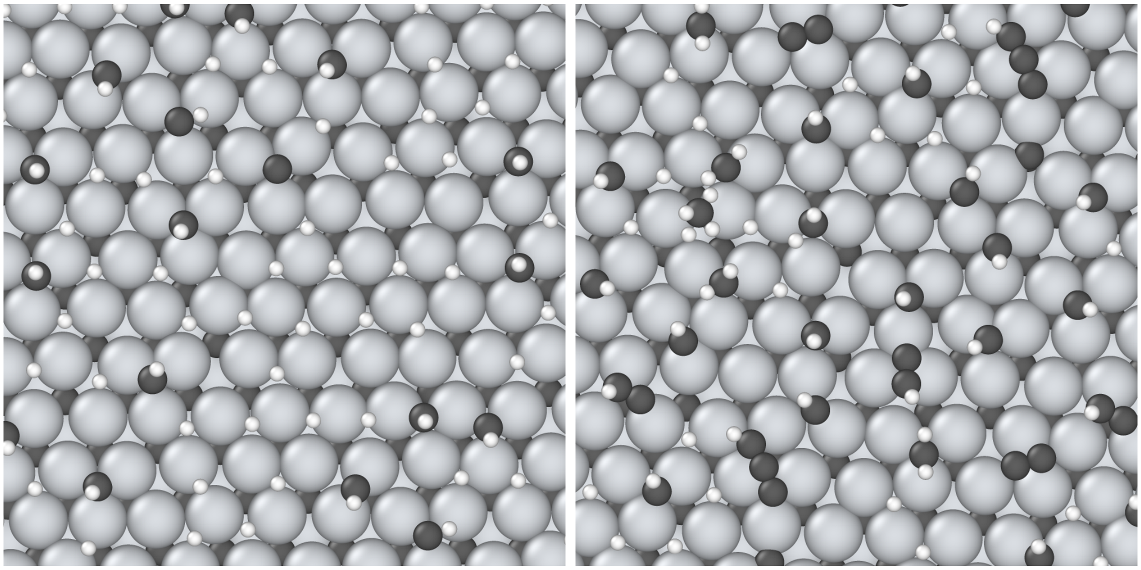

(a) Ti_8C_5(001) Ti terminated surface (b) Ti_8C_5(001) C terminated surface

Figure S23: Snapshots from an NVT-MD simulation at 1200 K of the Ti_8C_5(001) surface starting with 20 CH_4 molecules at t = 500 ps for (a) the Ti-terminated and (b) the C-terminated surface. The Ti-terminated surface has multiple hydrogen atoms and CH* from methane dissociation. At the C-terminated surface, we observed multiple C-C coupling intermediates along with intermediates from methane dissociation. The average C-C bond length of the coupling intermediates is 1.4 Å.

References

(S1) Kresse, G.; Hafner, J. Ab Initio Molecular Dynamics for Liquid Metals. *Phys. Rev. B* **1993**, *47*, 558–561.

(S2) Kresse, G.; Hafner, J. Ab Initio Molecular-Dynamics Simulation of the Liquid-Metal–Amorphous-Semiconductor Transition in Germanium. *Phys. Rev. B* **1994**, *49*, 14251–14269.

(S3) Kresse, G.; Furthmüller, J. Efficiency of ab-initio total energy calculations for metals and semiconductors using a plane-wave basis set. *Comput. Mater. Sci.* **1996**, *6*, 15–50.

(S4) Kresse, G.; Furthmüller, J. Efficient Iterative Schemes for Ab Initio Total-Energy Calculations Using a Plane-Wave Basis Set. *Phys. Rev. B* **1996**, *54*, 11169.

(S5) Blöchl, P. E. Projector Augmented-Wave Method. *Phys. Rev. B* **1994**, *50*, 17953.

(S6) Perdew, J. P.; Burke, K.; Ernzerhof, M. Generalized Gradient Approximation Made Simple. *Phys. Rev. Lett.* **1996**, *77*, 3865.

(S7) Zhang, Y.; Yang, W. Comment on "Generalized Gradient Approximation Made Simple". *Phys. Rev. Lett.* **1998**, *80*, 890–890.

(S8) Grimme, S.; Ehrlich, S.; Goerigk, L. Effect of the damping function in dispersion corrected density functional theory. *J. Comput. Chem.* **2011**, *32*, 1456–1465.

(S9) Wang, H.; Zhang, L.; Han, J.; E, W. DeePMD-Kit: A Deep Learning Package for Many-Body Potential Energy Representation and Molecular Dynamics. *Comput. Phys. Commun.* **2018**, *228*, 178–184.

(S10) Achar, S. K.; Bernasconi, L.; DeMaio, R. I.; Howard, K. R.; Johnson, J. K. In Silico Demonstration of Fast Anhydrous Proton Conduction on Graphanol. *ACS Appl. Mater. Interfaces* **2023**, *15*, 25873–25883, PMID: 37192530.

(S11) Zimmerman, P. M. Single-ended transition state finding with the growing string method. *J. Comput. Chem.* **2015**, *36*, 601–611.

(S12) Henkelman, G.; Uberuaga, B. P.; Jónsson, H. A climbing image nudged elastic band method for finding saddle points and minimum energy paths. *J. Chem. Phys.* **2000**, *113*, 9901–9904.

(S13) Henkelman, G.; Jónsson, H. Improved Tangent Estimate in the Nudged Elastic Band Method for Finding Minimum Energy Paths and Saddle Points. *J. Chem. Phys.* **2000**, *113*, 9978.

(S14) Henkelman, G.; Uberuaga, B. P.; Jónsson, H. A Climbing Image Nudged Elastic Band Method for Finding Saddle Points and Minimum Energy Paths. *J. Chem. Phys.* **2000**, *113*, 9901.

(S15) Sheppard, D.; Terrell, R.; Henkelman, G. Optimization Methods for Finding Minimum Energy Paths. *J. Chem. Phys.* **2008**, *128*, 134106.

(S16) Healy, J.; McInnes, L. Uniform manifold approximation and projection. *Nat. Rev. Methods Primers* **2024**, *4*, 83.

(S17) De, S.; Bartók, A. P.; Csányi, G.; Ceriotti, M. Comparing Molecules and Solids Across Structural and Alchemical space. *Phys. Chem. Chem. Phys.* **2016**, *18*, 13754–13769.

(S18) Fix, E. *Discriminatory Analysis: Nonparametric Discrimination, Consistency Properties*; USAF school of Aviation Medicine, 1985; Vol. 1.

(S19) Zhang, S.; Makoś, M. Z.; Jadrich, R. B.; Kraka, E.; Barros, K.; Nebgen, B. T.; Tretiak, S.; Isayev, O.; Lubbers, N.; Messerly, R. A. et al. Exploring the frontiers of condensed-phase chemistry with a general reactive machine learning potential. *Nat. Chem.* **2024**, *16*, 727–734.

(S20) Smith, J. S.; Isayev, O.; Roitberg, A. E. ANI-1: An Extensible Neural Network Potential with DFT Accuracy at Force Field Computational Cost. *Chem. Sci.* **2017**, *8*, 3192–3203.

(S21) Larsen, A. H.; Mortensen, J. J.; Blomqvist, J.; Castelli, I. E.; Christensen, R.; Dułak, M.; Friis, J.; Groves, M. N.; Hammer, B.; Hargus, C. et al. Atomic Simulation Environment—a Python Library for Working with Atoms. *J. Phys. Condens. Matter* **2017**, *29*, 273002.

(S22) Andersen, H. C. Molecular dynamics simulations at constant pressure and/or temperature. *J. Chem. Phys.* **1980**, *72*, 2384–2393.
